# Design, synthesis, and molecular docking studies of diphenylquinoxaline-6-carbohydrazide hybrids as potent α-glucosidase inhibitors

**DOI:** 10.1186/s13065-022-00848-4

**Published:** 2022-07-31

**Authors:** Keyvan Pedrood, Zahra Rezaei, Kimia Khavaninzadeh, Bagher Larijani, Aida Iraji, Samanesadat Hosseini, Somayeh Mojtabavi, Mehdi Dianatpour, Hossein Rastegar, Mohammad Ali Faramarzi, Haleh Hamedifar, Mir Hamed Hajimiri, Mohammad Mahdavi

**Affiliations:** 1 Endocrinology and Metabolism Research Center, Endocrinology and Metabolism Clinical Sciences Institute, Tehran University of Medical Sciences, Tehran, Iran; 2 Department of Medicinal Chemistry, School of Pharmacy, Iran University of Medical Sciences, Tehran, Iran; 3 Stem Cells Technology Research Center, Shiraz University of Medical Sciences, Shiraz, Iran; 4 Central Research Laboratory, Shiraz University of Medical Sciences, Shiraz, Iran; 5 Department of Pharmaceutical Chemistry, School of Pharmacy, Shahid Beheshti University of Medical Sciences, Tehran, Iran; 6 Department of Pharmaceutical Biotechnology, Faculty of Pharmacy & Biotechnology Research Center, Tehran University of Medical Sciences, Tehran, Iran; 7 Cosmetic Products Research Center, Iranian Food and Drug Administration, MOHE, Tehran, Iran; 8 CinnaGen Medical Biotechnology Research Center, Alborz University of Medical Sciences, Karaj, Iran; 9 Nano Alvand Company, Tehran University of Medical Sciences, Avicenna Tech Park, Tehran, Iran

**Keywords:** α-glucosidase inhibition, Type 2 diabetes, Quinoxaline, Hydrazone, Molecular docking

## Abstract

A novel series of diphenylquinoxaline-6-carbohydrazide hybrids **7a–o** were rationally designed and synthesized as anti-diabetic agents. All synthesized compounds **7a–o** were screened as possible α-glucosidase inhibitors and exhibited good inhibitory activity with IC_50_ values in the range of 110.6 ± 6.0 to 453.0 ± 4.7 µM in comparison with acarbose as the positive control (750.0 ± 10.5 µM). An exception in this trend came back to a compound **7k** with IC_50_ value > 750 µM. Furthermore, the most potent derivative **7e** bearing 3-fluorophenyl moiety was further explored by kinetic studies and showed the competitive type of inhibition. Additionally, the molecular docking of all derivatives was performed to get an insight into the binding mode of these derivatives within the active site of the enzyme. In silico assessments exhibited that **7e** was well occupied in the binding pocket of the enzyme through favorable interactions with residues, correlating to the experimental results.

**Supplementary Information:**

The online version contains supplementary material available at 10.1186/s13065-022-00848-4.

## Introduction

Diabetes mellitus (DM) can be construed as a chronic metabolic disorder identified by insistent hyperglycemia [1–3]. It can also be taken into consideration as the ailment of protein, fat, and carbohydrate metabolism resulting from failure in insulin secretion (type I diabetes), insulin dysfunction (type II diabetes), or both [4, 5]. Hyperglycemia, as the most stringent criterion of all types of diabetes, causes significant complications, including lipid metabolism disorders, kidney failure, neuropathy, and cardiovascular disorders [6]. Conducted by the International Diabetes Federation (IDF 2021), it was revealed that 537 million adults are globally afflicted with DM, and this number will increase dramatically to around 643 million by 2030 if no practical solution is discovered. There are several guidelines recommended for normalization of the blood glucose level, including controlled diet as well as physical exercise, which can be useful against a sedentary lifestyle [7]. Along the same line, to manage and control type II diabetes, one therapeutic way is to inhibit enzymes that convert carbohydrates into glucose [8–10].

In this regard, α-glucosidase is one of the most important enzyme found in the digestive system [11, 12]. The α-glucosidase presented on the brush border of human intestinal mucosal cells participates in the body's carbohydrate metabolism to convert oligosaccharides and disaccharides into monosaccharides by hydrolyzing the α-1,4-glycosidic bond [13–15]. Acarbose as a potent pseudo carbohydrate inhibitor of α-glucosidase and a-amylase reduces the breakdown of complex carbohydrates into monosaccharides such as glucose [16, 17]. However, long-term use may result in mild-to-moderate gastrointestinal side effects, flatulence, and diarrhea [18]. Other drugs such as biguanides (metformin), meglitinides, sulfonylureas, and thiazolidinediones are used as oral drugs to treat type II DM still associated with mentioned adverse side effects [19, 20]. Moreover, it is well documented that taking advantage of the other commercially available therapies controls only 36% of type II DM patients to achieve glycemic control [21]. As a result, the need for novel and safe α-glucosidase inhibitors to control the blood sugar level is critical [22–25].

Quinoxaline, also called benzo[b][1,4]diazine or benzopyrazine, and its derivatives are an integral part of medicinal chemistry owing to their wide range of biological activity, including antithrombotic, anti-tubercular, antitumor, antimalarial, antiplasmodial, antiprotozoal, AMPA receptor antagonist, and antiviral activities [26–31]. Besides, recent extensive studies have reported novel quinoxaline-based derivatives with high anti-α-glucosidase inhibitory potencies [32, 33].

Acyl hydrazone moiety has been known as a privileged structure in drug discovery due to its easy synthetic procedure via condensation of hydrazides and aldehydes or ketones under acid, base catalysis, or microwave irradiation [34]. This unique pharmacophore can participate in several interactions with the proposed targets through both hydrogen-bond acceptor and donor of amino-acid residues of enzyme binding site. Acylhydrazone exhibited a wide range of pharmacological potencies as antimicrobial [35], anticancer [36], analgesic [37], and anti-inflammatory(38) agents. Also, the potencies of acylhydrazone derivatives as tyrosinase [39, 40], acetylcholinesterase [41], BACE1 [42], and α-glucosidase [43] inhibitors were reported. Along the same line, Schiff bases are among the essential organic moiety with diverse biological activities such as urease, α-glucosidase, and β-glucuronidase inhibitory activities [44–49].

In the current study, novel biphenylquinoxaline derivatives bearing different acyl hydrazone were designed, synthesized, and evaluated against the α-glucosidase. The most potent compound was then subjected to kinetic study and molecular docking assessments.

## Results and discussion

### Designing

**Fig. 1:**
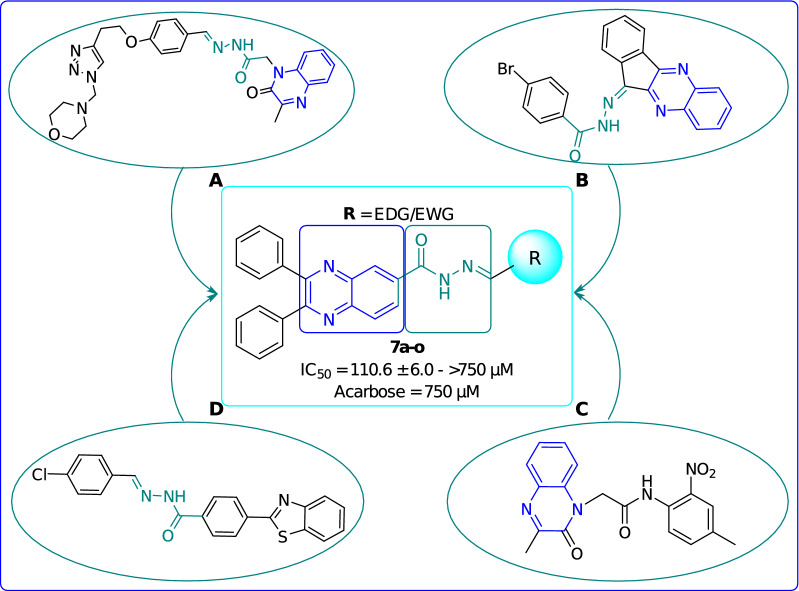
The rationale for the design of diphenylquinoxaline-6-carbohydrazide hybrids as new α-glucosidase inhibitors

There are a bunch of reports in the literature in which both quinoxaline and hydrazide–hydrazone scaffolds have shown potent α-glucosidase inhibitory activity. Take the example of the recent research, compound **A** (Fig. 1) containing both mentioned moieties exerted an IC_50_ value of 21.92 μg mL^−1^ in comparison with the standard acarbose (IC_50_ = 22.32 μg mL^−1^) as an effective α-glucosidase inhibitor [32]. In another similar research, compound **B** (IC_50_ = 22.67 ± 0.1 μmol mL^−1^ compared with the standard acarbose 38.25 ± 0.1 μmol mL^−1^) proved the potent effect of quinoxaline and acyl hydrazone moieties as the α-glucosidase inhibitor [15]. Moreover, concrete evidence supports that the compounds bearing each of these two scaffolds showed potent α-glucosidase inhibitory activity. In this context, compound **C** bearing quinoxaline ring with IC_50_ value of 83.78 ± 0.89 μmol mL^−1^ in comparison with the standard acarbose 72.58 ± 0.682 μmol mL^−1^ [15] and compound **D** with IC_50_ value of 83.78 ± 0.89 μmol mL^−1^ compared with the standard acarbose 72.58 ± 0.682 μmol mL^−1^ bearing hydrazide–hydrazone scaffold are good examples [47].

With this information in hand; herein, a series of diphenylquinoxaline-6-carbohydrazide hybrids as the novel agents against α-glucosidase were designed and synthesized. All derivatives were evaluated as α-glucosidase inhibitors in vitro and the most potent derivative in this group was subjected to a kinetic study to determine the type of inhibition. In addition, molecular docking studies of all derivatives were performed to get insight into the binding affinity and pose of these compounds within the enzyme binding site.

### Chemistry

**Scheme 1:**
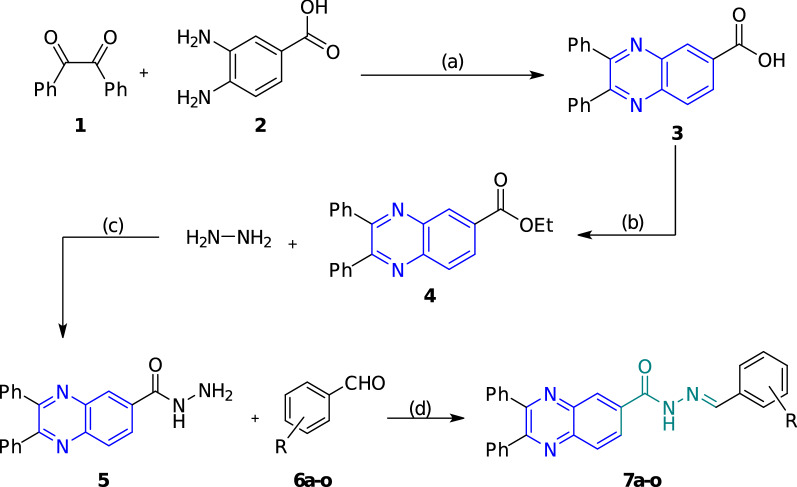
Outline for the synthesis of diphenylquinoxaline-2-carbohydrazide hybrids **7a–o,** reagents and conditions: **a** AcOH, 50 °C, 4–8 h; **b** dry EtOH, H_2_SO_4_, reflux, 12 h; **c** EtOH, room temperature, 16 h; **d** PTSA, EtOH, room temperature, 1 h

Scheme 1 represents the pathway for the synthesis of diphenylquinoxaline-6-carbohydrazide hybrids **7a–o.** As can be seen in this scheme, 2,3-diphenylquinoxaline-6-carboxylic acid **3** was synthesized through a reaction between commercial benzil **1** and 3,4-diaminobenzoic acid** 2** in acetic acid as a solvent in 50 °C. Then, the mentioned product **3** experienced an esterification reaction with dry ethanol as the solvent and reagent in the presence of a catalytic amount of sulfuric acid. Later on, the reaction of ethyl 2,3-diphenylquinoxaline-6-carboxylate **4** with hydrazine **8** resulted in the formation of 2,3-diphenylquinoxaline-6-carbohydrazide** 5** at room temperature. Finally, the reaction between the latter compound (**5**) and a wide range of aldehydes **6a-o** led to the formation of the final products **7a–o**. The latter derivatives (**7a-o**) were fully characterized by ^1^H NMR, ^13^C NMR, FT-IR, and elemental analysis.

### α-glucosidase inhibitory activity

In this project, which is aimed to develop new α-glucosidase inhibitors, all aromatic carbohydrazide derivatives **7a–o** were screened. The synthetic compounds showed a varying degree of α-glucosidase inhibition with IC_50_ values in the range of 110.6 to more than 750 µM (*Table **1*).

**Table 1:** In vitro α-glucosidase inhibitory activities of compounds **7a–o**


Compounds	R	IC_50_ (µM) ^a,b^
7a	C_6_H_5_	154.8 ± 3.0
7b	2–NO_2_–C_6_H_4_	175.0 ± 5.9
7c	3–NO_2_–C_6_H_4_	278.6 ± 5.8
7d	4–NO_2_–C_6_H_4_	239.7 ± 7.5
7e	3–F–C_6_H_4_	110.6 ± 6.0
7f	4–Cl–C_6_H_4_	260.3 ± 4.5
7g	4–OMe–C_6_H_4_	319.7 ± 4.9
7h	2–NO_2_–3–OMe–C_6_H_3_	305.5 ± 7.2
7i	2–Cl–5–NO_2_–C_6_H_3_	230.8 ± 5.5
7j	3–OMe–4–OH–C_6_H_3_	358.2 ± 6.1
7k	3,4,5–trimethoxy–C_6_H_2_	> 750
7l	3–OPh–C_6_H_4_	453.0 ± 4.7
7m	5-Nitrobenzo[d][1,3]dioxole	353.7 ± 3.5
7n	Naphthyl	388.1 ± 6.5
7o	Thiophene	145.4 ± 5.0
Acarbose	–	750.0 ± 10.5

^a^Values are the mean ± SEM. All experiments were performed at least three times

^b^According to the ANOVA test followed by Tukey post hoc, all derivatives exhibited significant differences (*p*-value < 0.05) compared to other compounds except **7a** *vs* **7o**, **7d** *vs* **7i**, **7g** vs **7h**, and **7j** *vs* **7n**

According to the results of the investigation, **7a** being phenyl moiety exhibited an IC_50_ value of 154.8 ± 3.0 µM. Next, the effect of compounds with electron-withdrawing groups was investigated, and it was shown that the presence of all of the electron-withdrawing groups (except fluorine) caused a decrease in inhibitory potencies. In detail, the appearance of the nitrophenyl group at the *ortho* position (**7b**) had the least negative impact on the inhibition, followed by **7d** (R = 4–NO_2_–C_6_H_4_) ˃ **7c** (R = 3–NO_2_–C_6_H_4_). This order of potency could attribute to both the power of inductive and resonance effects of these moieties in the mentioned position.

Compound **7f** bearing chlorophenyl substitution (as an electron-withdrawing group) at *para* position showed less inhibitory effect than **7d** (in which the nitro moiety is in *para* position). The reason can be ascribed to the differences in electronegativity of the mentioned substitutions. Among all electron-withdrawing groups, just compound **7e** possessing fluorophenyl substituent at the *meta*-position was found to show potent inhibitory activity which could be due to electron-withdrawing potencies as well equality in size with H- substituent (**7a**). **7 g** derivative bearing *para-*methoxyphenyl substituent as electron-donating groups showed an IC_50_ value of 305 µM, which was a higher value than **7a** as unsubstituted derivative as well as all electron-withdrawing substituted compounds.

Also, the presence of multi-substitution groups (**7h**, **7i**, **7j**, **7k**) caused a destructive effect on α-glucosidase inhibition compared to unsubstituted derivative (**7a**).

Further, the effect of ring replacements was also evaluated. Results disclosed that bulky ring substitutions such as phenoxy phenyl (**7l**), 5-nitrobenzodioxole (**7m**), and naphthyl (**7n**) reduced the inhibitory activity significantly compared to phenyl counterpart (**7a**). Noteworthy, the replacement of phenyl ring with thiophene moiety **7o**, as a classical bioisostere of phenyl, slightly improved the α-glucosidase inhibition. The comparative betterment achieved in the effect of thiophene moiety can be referred to as the more lipophilicity of thiophene in comparison with phenyl moiety.

Overall, the presence of one substituent on the phenyl, whether electron-withdrawing or donating groups (compounds **7b**-**g**) or even multi-substitutions, resulted in a relative decrease in acquired effects. Also, the presence of bulky rings showed a further negative impact on pharmacological activity. It seems that the pocket of the receptor has limited space to bind to the derivatives and regarding that, the backbone of the designed structure is bulky and spacious, the presence of small substitutions such as phenyl, fluorophenyl and thiophene moieties are more favorable.

### Enzyme kinetic studies

**Fig. 2:**
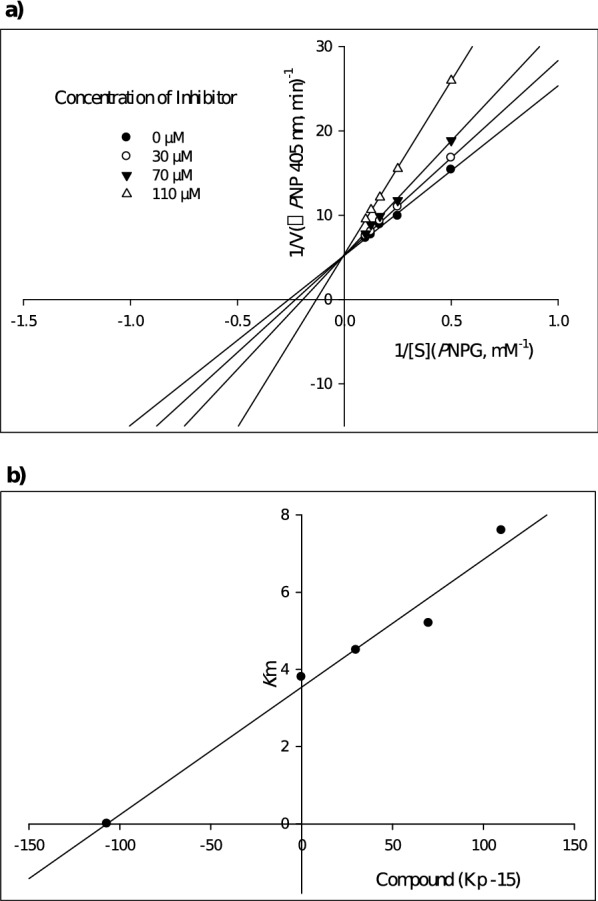
Kinetics of α-glucosidase inhibition by sample **7e**. **a** The Lineweaver—Burk plot in the absence and presence of different concentrations of sample **7e**; **b** The secondary plot between *K*_m_ and various concentrations of sample **7e**

According to Fig. 2A, the Lineweaver–Burk plot showed that the *K*_m_ gradually increased and *V*_*max*_ remained unchanged with increasing inhibitor concentration indicating a competitive inhibition. The results show sample **7e** bind to the active site on the enzyme and compete with the substrate for binding to the active site. Furthermore, the plot of the *K*_m_ versus different concentrations of inhibitor gave an estimate of the inhibition constant, *K*_i_ of 107 µM (Fig. 2B).

### Docking analyses

**Fig. 3:**
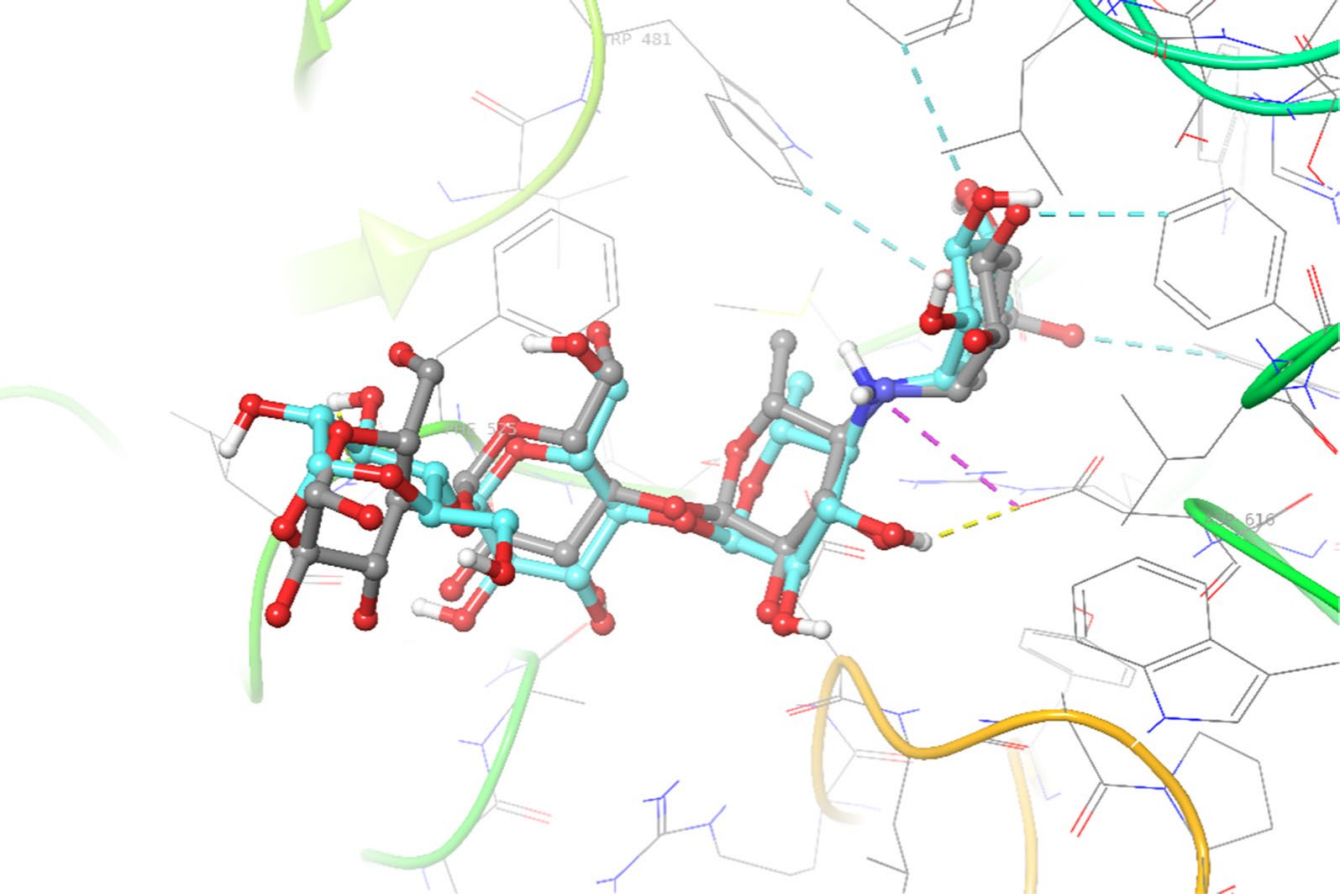
Superimpose structure of crystallographic ligand (blue) and docked acarbose (gray) in the active site of the α-glucosidase enzyme

Next, the molecular docking studies of all derivatives were performed. In the first step, to properly predict the binding pose of derivatives within the active site, the redocking process of acarbose (as a crystallographic ligand) with human lysosomal acid-alpha-glucosidase was performed using induce fit docking of the Schrodinger package. Alignment of the best pose of acarbose in the active site of α-glucosidase and crystallographic ligand recorded an RMSD value of 1.73 Å (Fig. 3).

Next, the same procedure was then applied for the docking of all derivatives, and the results are summarized in Table 2. The molecular docking study showed the binding energy of acarbose as a native ligand was − 6.143 kcal/mol while the glide score value of **7a –o** ranges from − 2.207 to − 5.802 kcal/mol. As can be seen the most potent derivative in in vitro assay was **7e** (IC_50_ = 110.6 ± 6.0 µM) > **7o** (IC_50_ = 145.4 ± 5.0 µM) > **7a** (IC_50_ = 154.8 ± 3.0 µM) > **7b** (IC_50_ = 175.0 ± 5.9 µM) exhibited the best in silico results with docking score value of − 5.802, − 5.493, − 5.690 and − 5.520 kcal/mol, respectively. Similarly, the least active derivatives **7k** demonstrated the worse result with a dock value of -2.207 kcal/mol. The violation of IC_50_ values and in silico results came back to **7h** (R = 2–NO_2_–3–OMe–C_6_H_3_) with moderate inhibition (IC_50_ = 305.5 ± 7.2) and a good glide score value.

**Table 2:** Docking results of synthesized compounds within the binding pocket of the α-glucosidase

Compounds	Glide score	Type of interaction	Moiety	Residue
7a	− 5.690	Pi-pi-stacked Pi-pi-stacked Pi-pi-stacked Pi-pi-stacked -bound	Phenyl Phenyl Phenyl Quinoxaline Amide	Trp376 Trp481 Phe649 Phe525 Asp282
7b	− 5.520	Salt bridge Pi-pi-stacked Pi-pi-stacked H-bound	Nitro Nitrophenyl Nitrophenyl Amide	Asp282 Trp481 Phe525 Asp282
7c	− 5.756	Aromatic H-bound Aromatic H-bound Aromatic H-bound Aromatic H-bound H-bound	Phenyl Phenyl Phenyl Quinoxaline Quinoxaline	Asp404 Asp518 Asp616 Trp481 Asp282
7d	− 5.175	Aromatic H-bound Aromatic H-bound Aromatic H-bound Aromatic H-bound H-bound	Phenyl Phenyl Quinoxaline Quinoxaline Quinoxaline	Asp404 Asp616 Trp481 Asp518 Asp282
7e	− 5.802	Aromatic H-bound Aromatic H-bound Aromatic H-bound Aromatic H-bound H-bound	Phenyl Phenyl Quinoxaline Quinoxaline Quinoxaline	Asp404 Asp616 Trp481 Phe525 Trp481
7f	− 5.173	Aromatic H-bound Aromatic H-bound Aromatic H-bound Pi-cation Aromatic H-bound H-bound	Phenyl Phenyl Phenyl 4-Clphenyl Quinoxaline Amide	Asp518 Asp616 Phe649 Arg600 Asp282 Asp282
7g	− 3.516	H-bound Aromatic H-bound	Amide 4-meophenyl	Asp282 Trp516
7h	− 5.681	Aromatic H-bound Aromatic H-bound Aromatic H-bound Pi-cation H-bound	Phenyl Nitrophenyl Nitrophenyl Nitrophenyl Amide	Trp618 Asp616 Trp481 Asp616 Asp282
7i	− 5.360	Aromatic H-bound Aromatic H-bound Halogen Pi-cation Pi-cation H-bound	Phenyl Phenyl Chlorophenyl Nitrophenyl Nitrophenyl Amide	Trp481 Trp481 Trp481 Asp518 Asp616 Asp282
7j	− 3.641	H-bound H-bound	Amide OH	Asp282 Asp404
7k	− 2.207	H-bound	Phenyl	Asp616
7l	− 4.028	H-bound H-bound	Amide Amide	Asp282 Ala284
7m	− 4.763	Pi-cation Pi-pi- stacked Pi-pi- stacked	NO_2_ Quinoxaline Quinoxaline	Arg281 Phe525 Trp481
7n	− 3.886	Pi-cation Pi-pi- stacked Pi-pi- stacked	H-bound Quinoxaline Quinoxaline	Ars282 Phe649 Trp376
7o	− 5.493	H-bound H-bound Salt bridge Pi-pi- stacked Pi-pi- stacked Aromatic H-bound	Amide Amide Amide Thiophene Quinoxaline Phenyl	Arg411 Arg411 Arg411 Trp481 Trp481 Asp616
Acarbose	− 6.143	H-bound Salt bridge H-bound H-bound	OH NH OH OH	Asp616 Asp616 Asp518 Phe525

**Fig. 4:**
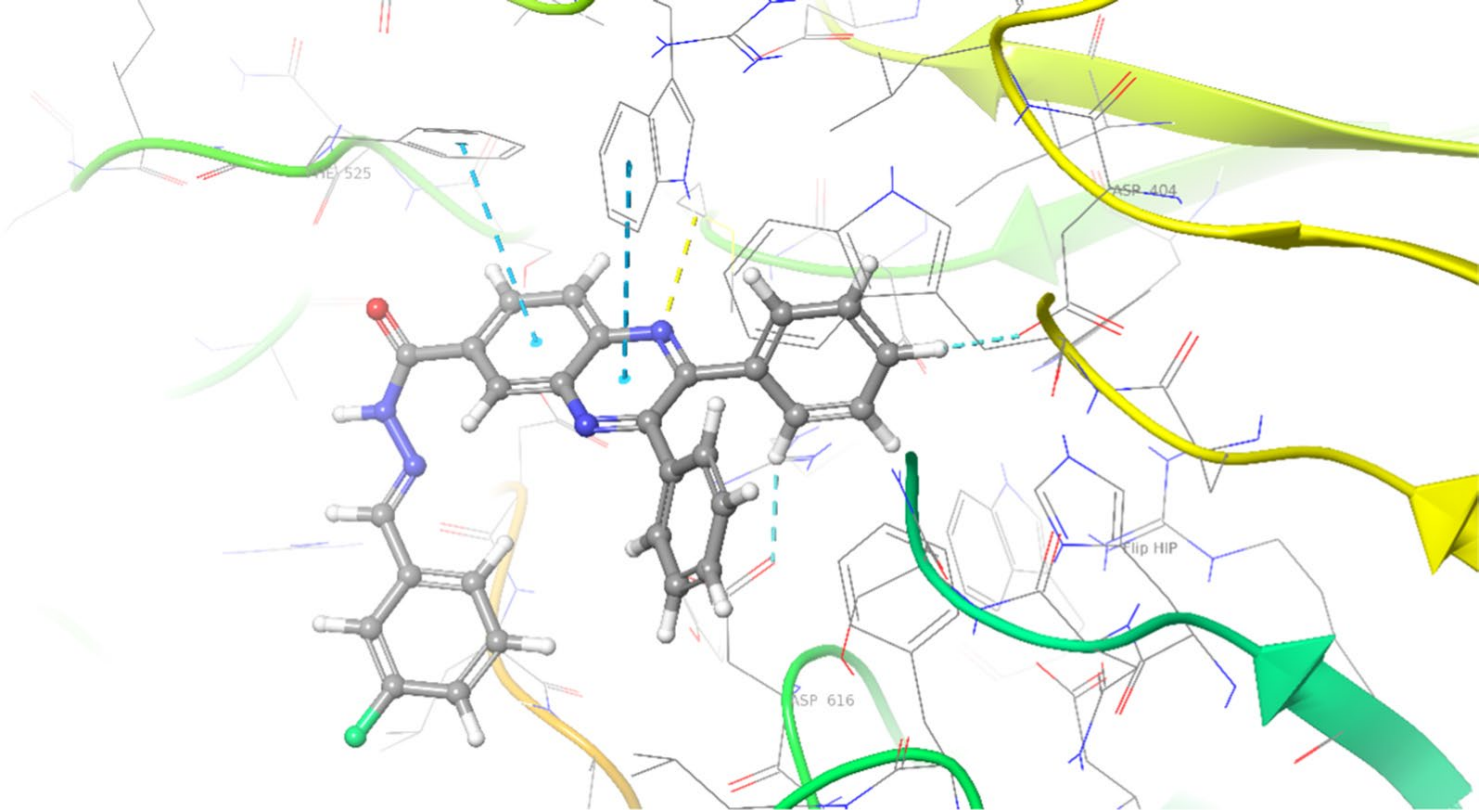
3D interaction pattern of compounds **7e** (most potent derivative) within the α-glucosidase active site

**Fig. 5:**
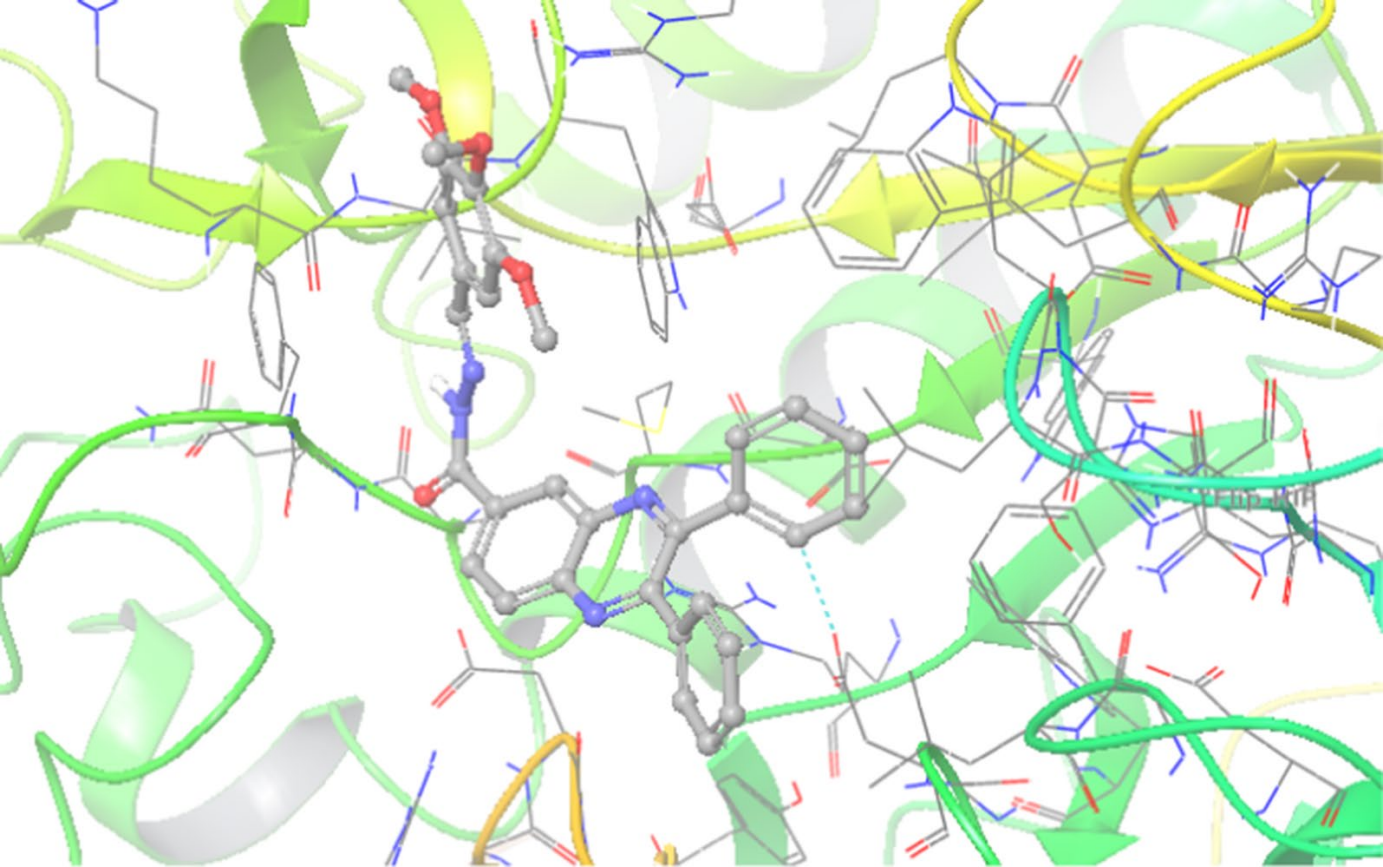
3D interaction pattern of compounds **7k** (inactive derivative) within the α-glucosidase active site

3D interaction pattern of **7e** (Fig. 4) showed quinoxaline ring made one hydrogen bound interaction with Trp481 and two pi-pi stacked interactions with Trp481 and Phe525, respectively. Phenyl ring also recorded aromatic H-bound interaction with Asp616 and Asp404. However, the 3D interaction pattern of **7k** (inactive derivative) exhibited interesting results so the presence of three methoxy groups changes the orientation of the molecular within the binding pocket so that it cannot fit into the binding site and can not participate in critical interaction with the enzyme (Fig. 5).

## Conclusion

In conclusion, a novel series of diphenylquinoxaline-6-carbohydrazide hybrids **7a–o** as the new anti-diabetic agents with α-glucosidase inhibitory potential were designed and synthesized. These novel compounds exhibited good α-glucosidase inhibitory activity with IC_50_ values in the range of 110.6 ± 6.0 to ˃750 µM in comparison with acarbose (IC_50_ = 750.0 ± 10.5 µM) as the positive control. Compound **7e**, as the most potent derivative, was further investigated, and the kinetic studies showed that the type of inhibition for compound **7e** is competitive, which means it competed with the substrate to attach to the active site of the enzyme. Molecular docking studies of **7e** within the α-glucosidase active site demonstrated that this molecule fitted well into the α-glucosidase binding pocket and showed hydrogen, aromatic hydrogen bond interactions. Thus, the derivatives appear to be an ideal candidate for initiating lead anti-DM drug discovery.

## Experimental

### Chemistry

The measured data on melting points were evaluated on a Kofler hot stage apparatus and were uncorrected for most derivatives. The NMR (^1^H and ^13^C) and IR spectra were gained by employing Bruker 300-NMR and ALPHA FT-IR spectrometer on KBr disks, respectively. The chemical reagents were obtained from Aldrich and Merck as well. Elemental analyses were also performed on an Elementar Analysensystem GmbH VarioEL CHNS mode. Moreover, the Spectroscopic data of final products, including ^1^H and ^13^C NMR, are available in the supporting information.

#### General procedure for the synthesis of 2,3-diphenylquinoxaline-6-carboxylic acid 3

The mixture of equivalent amounts of benzil **1** (5 mmol, 1.05 g) and 3,4-diaminobenzoic acid **2** (5 mmol, 0.76 g) was stirred in glacial acetic acid (20 ml) at 50 °C for 4–8 h. After completion of the reaction (monitored by the TLC), the participated product **3** was filtrated and purified by ethanol or ethyl acetate[42, 50].

#### General procedure for the synthesis of ethyl 2,3-diphenylquinoxaline-6-carboxylate 4

The 2,3‐diphenylquinoxaline-6-carboxylic acid **3** (5 mmol) was poured in dry ethanol (20 ml), and H_2_SO_4_ was added to the medium. The mixture was refluxed for 12 h, and the white solid of the desired product **4** was filtered off after pouring the mixture into the water.

#### General procedure for the synthesis of 2,3-diphenylquinoxaline-6-carbohydrazide 5.

The mixture of 2,3-diphenylquinoxaline-6-carboxylate **4** (5 mmol, 1.05 g) and hydrazine (15 ml) was stirred in ethanol (20 ml) at the ambient temperature for 16 h. After completion of the reaction (monitored by the TLC), the participated product **5** was filtrated and purified by ethanol or ethyl acetate.

#### General procedure for the synthesis of diphenylquinoxaline-2-carbohydrazide derivatives *7a–o*

A mixture of 2,3-diphenylquinoxaline-6-carbohydrazide **5** (1 mmol) and appropriate benzaldehydes (**6a–o**) (1 mmol) in the presence of a catalytic amount of *para*-toluenesulfonic acid (PTSA) in ethanol was stirred at room temperature for 1 h. Then, the mixture was extracted with ethyl acetate, dried over anhydrous sodium sulfate, filtered and the solvent was evaporated. The residue was purified by column chromatography to give the final products (**7a–o**) (Additional file 1).

#### N'-benzylidene-2,3-diphenylquinoxaline-6-carbohydrazide *7a*

White solid. Yield: 77%. Mp 230–232 °C; IR (KBr): υ (cm^−1^) = 3213, 3058, 1677, 1555. ^1^H NMR (301 MHz, DMSO-*d*_6_) δ 12.18 (s, 1H), 8.79 (d, *J* = 1.8 Hz, 1H), 8.53 (s, 1H), 8.33 (d, *J* = 8.7 Hz, 1H), 8.21 (d, *J* = 8.8 Hz, 1H), 7.67 (d, *J* = 7.7 Hz, 2H), 7.52 – 7.40 (m, 5H), 7.40 – 7.32 (m, 5H), 7.27 (t, *J* = 7.7 Hz, 2H), 7.16 (t, *J* = 7.8 Hz, 1H). ^13^C NMR (76 MHz, DMSO-*d*_6_) δ 162.32, 154.77, 154.37, 148.93, 142.25, 140.48, 140.20, 138.96, 138.92, 134.79, 132.03, 130.25, 130.17, 129.90, 129.55, 129.46, 128.56, 127.66 ppm. Anal. calcd. For C_28_H_20_N_4_O: C, 78.49; H, 4.70; N, 13.08. Found: C, 78.41; H, 4.63; N, 13.16.

#### N'-(2-nitrobenzylidene)-2,3-diphenylquinoxaline-6-carbohydrazide *7b*

Pale yellow solid. Yield: 90%. Mp > 250 °C; IR (KBr): υ (cm^−1^) = 3193, 3065, 1692, 1617, 1530. ^1^H NMR (301 MHz, DMSO-*d*_6_) δ 12.55 (s, 1H), 8.98 (s, 1H), 8.83 (s, 1H), 8.34 (d, *J* = 8.8 Hz, 1H), 8.27–8.13 (m, 2H), 8.09 (d, *J* = 8.2 Hz, 1H), 7.83 (t, *J* = 7.7 Hz, 1H), 7.68 (t, *J* = 7.8 Hz, 1H), 7.61 – 7.43 (m, 5H), 7.43 – 7.29 (m, 5H). ^13^C NMR (76 MHz, DMSO-*d*_6_) δ 162.51, 154.96, 154.47, 148.70, 144.00, 142.35, 140.16, 138.94, 138.91, 134.18, 131.21, 130.25, 130.17, 129.57, 129.49, 129.13, 128.90, 128.56, 128.40, 125.15 ppm. Anal. calcd. For C_28_H_19_N_5_O_3_: C, 71.03; H, 4.04; N, 14.79. Found C, 71.14; H, 3.96; N, 14.68.

#### N'-(3-nitrobenzylidene)-2,3-diphenylquinoxaline-6-carbohydrazide *7c*

Pale yellow solid. Yield: 91%. Mp > 250 °C; IR (KBr): υ (cm^−1^) = 3196, 3071, 1696, 1613, 1523. ^1^H NMR (301 MHz, DMSO-*d*_6_) δ 12.45 (s, 1H), 8.79 (d, *J* = 1.9 Hz, 1H), 8.62 (s, 1H), 8.53 (s, 1H), 8.33 (dd, *J* = 8.6, 1.8 Hz, 1H), 8.27 – 8.18 (m, 2H), 8.15 (d, *J* = 7.8 Hz, 1H), 7.73 (t, *J* = 7.9 Hz, 1H), 7.56 – 7.48 (m, 5H), 7.46 – 7.34 (m, 5H). ^13^C NMR (76 MHz, DMSO-*d*_6_) δ 162.25, 155.11, 154.62, 150.91, 142.32, 141.45, 140.42, 140.14, 134.05, 132.22, 130.26, 130.11, 129.69, 129.58, 129.46, 129.26, 128.86, 128.85, 128.48, 127.17, 124.72 ppm. Anal. calcd. For C_28_H_19_N_5_O_3_: C, 71.03; H, 4.04; N, 14.79. Found C, 71.15; H, 4.12; N, 14.66.

#### N'-(4-nitrobenzylidene)-2,3-diphenylquinoxaline-6-carbohydrazide *7d*

Pale yellow solid. Yield: 92%. Mp > 250 °C; IR (KBr): υ (cm^−1^) = 3194, 3074, 1691, 1616, 1529. ^1^H NMR (301 MHz, DMSO-*d*_6_) δ 12.45 (s, 1H), 8.76 (d, *J* = 2.0 Hz, 1H), 8.58 (s, 1H), 8.31 (dd, *J* = 8.6, 1.8 Hz, 1H), 8.27 – 8.17 (m, 3H), 7.96 (d, *J* = 8.4 Hz, 2H), 7.56 – 7.44 (m, 5H), 7.43 – 7.33 (m, 5H). ^13^C NMR (76 MHz, DMSO-*d*_6_) δ 161.45, 154.75, 154.34, 148.82, 142.21, 140.30, 138.90, 134.86, 134.28, 130.14, 129.53, 129.41, 129.39, 129.25, 128.50, 127.32, 126.06, 124.93 ppm. Anal. calcd. For C_28_H_19_N_5_O_3_: C, 71.03; H, 4.04; N, 14.79. Found C, 71.12; H, 4.08; N, 14.72.

#### N'-(3-fluorobenzylidene)-2,3-diphenylquinoxaline-6-carbohydrazide *7e*

White solid. Yield: 84%. Mp > 250 °C; IR (KBr): υ (cm^−1^) = 3228, 3073, 1711, 1557, 1248. ^1^H NMR (301 MHz, DMSO-*d*_6_) δ 12.31 (s, 1H), 8.78 (s, 1H), 8.54 (s, 1H), 8.31 (d, *J* = 8.7 Hz, 1H), 8.18 (d, *J* = 8.6 Hz, 1H), 7.61 – 7.40 (m, 8H), 7.39 – 7.28 (m, 5H), 7.24 (td, *J* = 8.5, 2.7 Hz, 1H). ^13^C NMR (76 MHz, DMSO-*d*_6_) δ 162.84 (*J*_*C*–F_ = 245.2 Hz), 162.43, 154.78, 154.33, 147.39 (*J*_*C*–F_ = 2.2 Hz), 142.27, 140.14, 138.93, 138.89, 137.27 (*J*_*C*–F_ = 7.9 Hz), 134.42, 131.31 (*J*_*C*–F_ = 7.4 Hz), 130.24, 130.16, 129.53, 128.79, 128.53, 124.03 (*J*_*C*–F_ = 1.6 Hz), 117.31 (*J*_*C*–F_ = 21.5 Hz), 113.48 (*J*_*C*–F_ = 22.7 Hz) ppm. Anal. calcd. For C_28_H_19_FN_4_O: C, 75.32; H, 4.29; N, 12.55. Found: C, 75.39; H, 4.06; N, 12.61.

#### N'-(4-chlorobenzylidene)-2,3-diphenylquinoxaline-6-carbohydrazide *7f*

White solid. Yield: 90%. Mp 234–238 °C; IR (KBr): υ (cm^−1^) = 3422, 1781, 1688, 720. ^1^H NMR (301 MHz, DMSO-*d*_6_) δ 12.30 (s, 1H), 8.79 (d, *J* = 1.9 Hz, 1H), 8.54 (s, 1H), 8.34 (d, *J* = 8.8 Hz, 1H), 8.26 (d, *J* = 8.7 Hz, 1H), 7.80 (d, *J* = 8.2 Hz, 2H), 7.58 – 7.42 (m, 7H), 7.42 – 7.31 (m, 5H). ^13^C NMR (76 MHz, DMSO-*d*_6_) δ 162.50, 154.96, 154.52, 147.58, 144.14, 142.30, 140.18, 138.95, 138.91, 135.15, 134.65, 133.64, 130.25, 130.17, 129.44, 129.31, 128.76, 128.60 ppm. Anal. calcd. For C_28_H_19_ClN_4_O: C, 72.65; H, 4.14; N, 12.10. Found: C, 72.53; H, 3.97, 12.27.

#### N'-(4-methoxybenzylidene)-2,3-diphenylquinoxaline-6-carbohydrazide *7 g*

White solid. Yield: 81%. Mp > 250 °C; IR (KBr): υ (cm^−1^) = 3199, 3054, 1697, 1550. ^1^H NMR (301 MHz, DMSO-*d*_6_) δ 12.11 (s, 1H), 8.78 (s, 1H), 8.51 (s, 1H), 8.32 (dd, *J* = 8.5, 1.8 Hz, 1H), 8.21 (d, *J* = 8.5 Hz, 1H), 7.71 (d, *J* = 8.4 Hz, 2H), 7.56 – 7.40 (m, 5H), 7.39 – 7.27 (m, 5H), 7.01 (d, *J* = 8.3 Hz, 2H), 3.59 (s, 3H). ^13^C NMR (76 MHz, DMSO-*d*_6_) δ 162.24, 161.39, 154.73, 154.34, 148.81, 142.20, 140.19, 138.96, 138.92, 134.86, 130.24, 130.16, 129.52, 129.45, 129.29, 128.55, 127.27, 114.77, 55.74 ppm. Anal. calcd. For C_29_H_22_N_4_O_2_: C, 75.97; H, 4.84; N, 12.22. Found: C, 76.11; H, 4.73; N, 12.16.

#### N'-(3-methoxy-2-nitrobenzylidene)-2,3-diphenylquinoxaline-6-carbohydrazide *7h*

Pale yellow solid. Yield: 86%. Mp > 250 °C; IR (KBr): υ (cm^−1^) = 3191, 3032, 1707, 1586, 1515. ^1^H NMR (301 MHz, DMSO-*d*_6_) δ 12.42 (s, 1H), 8.79 (d, *J* = 1.9 Hz, 1H), 8.48 (s, 1H), 8.33 (dd, J = 8.7, 1.8 Hz, 1H), 8.23 (d, *J* = 8.7 Hz, 1H), 7.71 – 7.60 (m, 2H), 7.56 – 7.47 (m, 5H), 7.47 – 7.31 (m, 6H), 3.94 (s, 3H). ^13^C NMR (76 MHz, DMSO-*d*_6_) δ 162.31, 155.01, 154.53, 150.86, 142.38, 141.55, 140.38, 140.15, 138.94, 138.90, 134.09, 132.27, 130.25, 130.17, 129.50, 129.32, 128.83, 128.57, 127.12, 118.69, 57.30 ppm. Anal. calcd. For C_29_H_21_N_5_O_4_: C, 69.18; H, 4.20; N, 13.91. Found: C, 69.29; H, 4.28; N, 13.80.

#### N'-(2-chloro-5-nitrobenzylidene)-2,3-diphenylquinoxaline-6-carbohydrazide *7i*

Pale yellow solid. Yield: 88%. Mp > 250 °C; IR (KBr): υ (cm^−1^) = 3192, 3039, 1711, 1593, 1521. ^1^H NMR (301 MHz, DMSO-*d*_6_) δ 11.89 (s, 1H), 8.77 (s, 1H), 8.51 (s, 1H), 7.44 – 7.41 (m, 2H), 8.22 (d, *J* = 8.7 Hz, 1H), 8.02 (d, *J* = 7.3 Hz, 1H), 7.85 – 7.82 (m, 4H), 7.73 (d, *J* = 7.3 Hz, 1H), 7.42 – 7.34 (m, 6H). ^13^C NMR (76 MHz, DMSO-*d*_6_) δ 164.03, 154.90, 154.31, 148.52, 143.95, 142.15, 140.07, 139.99, 138.78, 134.14, 131.21, 130.26, 130.15, 129.61, 129.57, 129.41, 129.13, 128.90, 128.56, 128.32, 125.12, 124.15 ppm. Anal. calcd. For C_28_H_18_ClN_5_O_3_: C, 66.21; H, 3.57; N, 13.79. Found: C, 66.09; H, 3.42; N, 13.91.

#### N'-(4-hydroxy-3-methoxybenzylidene)-2,3-diphenylquinoxaline-6-carbohydrazide *7j*

White solid. Yield: 73%. Mp > 250 °C; IR (KBr): υ (cm^−1^) = 3232, 3073, 1696, 1579, 1288. ^1^H NMR (301 MHz, DMSO-*d*_6_) δ 12.12 (s, 1H), 9.72 (s, 1H), 8.70 (s, 1H), 8.46 (s, 1H), 8.26 (d, *J* = 8.8 Hz, 1H), 8.09 (d, *J* = 8.7 Hz, 1H), 7.51 – 7.36 (m, 5H), 7.35 – 7.20 (m, 6H), 7.10 (d, *J* = 8.1 Hz, 1H), 6.88 (d, *J* = 8.0 Hz, 1H), 3.83 (s, 3H). ^13^C NMR (76 MHz, DMSO-*d*_6_) δ 162.51, 154.54, 154.10, 150.51, 149.66, 148.55, 142.12, 140.10, 138.81, 138.79, 134.66, 130.19, 130.09, 129.39, 128.56, 128.44, 126.09, 123.10, 115.83, 109.31, 55.98 ppm. Anal. calcd. For C_29_H_22_N_4_O_3_: C, 73.40; H, 4.67; N, 11.81. Found: C, 73.48; H, 4.56; N, 11.84.

#### 3-diphenyl-N'-(3,4,5-trimethoxybenzylidene)quinoxaline-6-carbohydrazide *7k*

White solid. Yield: 70%. Mp > 250 °C; IR (KBr): υ (cm^−1^) = 3298, 3207, 3065, 1660, 1605. ^1^H NMR (301 MHz, DMSO-*d*_6_) δ 12.24 (s, 1H), 8.76 (s, 1H), 8.49 (s, 1H), 8.31 (d, *J* = 8.8 Hz, 1H), 8.19 (d, *J* = 8.7 Hz, 1H), 7.56 – 7.41 (m, 5H), 7.40 – 7.29 (m, 5H), 7.06 (s, 2H), 3.84 (s, 6H), 3.72 (s, 3H). ^13^C NMR (76 MHz, DMSO-*d*_6_) δ 162.51, 154.74, 154.32, 153.64, 148.96, 142.23, 140.14, 139.81, 138.91, 138.89, 134.71, 130.22, 130.14, 129.53, 129.45, 128.69, 128.52, 104.88, 60.57, 56.37 ppm. Anal. calcd. For C_31_H_26_N_4_O_4_: C, 71.80; H, 5.05; N, 10.80. Found: C, 71.69; H, 5.19; N, 10.77.

#### N'-(3-phenoxybenzylidene)-2,3-diphenylquinoxaline-6-carbohydrazide *7 l*

White solid. Yield: 73%. Mp > 250 °C; IR (KBr): υ (cm^−1^) = 3230, 3063, 1711, 1568. ^1^H NMR (301 MHz, DMSO-*d*_6_) δ 12.27 (s, 1H), 8.79 (d, *J* = 1.8 Hz, 1H), 8.55 (s, 1H), 8.33 (dd, *J* = 8.8, 1.9 Hz, 1H), 8.25 (d, *J* = 8.7 Hz, 1H), 7.57 – 7.46 (m, 5H), 7.45 – 7.32 (m, 10H), 7.21 (d, *J* = 7.4 Hz, 1H), 7.19 – 7.04 (m, 3H). ^13^C NMR (76 MHz, DMSO-*d*_6_) δ 162.45, 157.77, 156.77, 154.91, 154.48, 148.20, 142.30, 140.18, 138.96, 138.92, 136.74, 134.64, 131.05, 130.67, 130.25, 130.18, 129.64, 129.50, 128.76, 128.59, 124.33, 123.36, 119.47, 116.26 ppm. Anal. calcd. For C_34_H_24_N_4_O_2_: C, 78.44; H, 4.65; N, 10.76. Found: C, 78.39; H, 4.81; N, 10.83.

#### N'-((6-nitrobenzo[d][1,3]dioxol-5-yl)methylene)-2,3-diphenylquinoxaline-6-carbohydrazide *7 m*

Pale yellow solid. Yield: 89%. Mp > 250 °C; IR (KBr): υ (cm^−1^) = 3214, 3077, 1688, 1565, 1522. ^1^H NMR (301 MHz, DMSO-*d*_6_) δ 12.23 (s, 1H), 8.76 (s, 1H), 8.48 (s, 1H), 8.30 (d, *J* = 8.9 Hz, 1H), 8.21 (d, *J* = 8.8 Hz, 1H), 8.01 (s, 1H), 7.51 – 7.44 (m, 5H), 7.40 – 7.29 (m, 5H), 7.16 (s, 1H), 5.97 (s, 2H). ^13^C NMR (76 MHz, DMSO-*d*_6_) δ 162.66, 158.76, 158.17, 154.57, 153.68, 149.49, 148.25, 142.12, 140.10, 138.38, 134.19, 130.19, 130.09, 129.39, 128.44, 127.92, 125.50, 122.49, 115.93, 110.85, 100.65 ppm. Anal. calcd. For C_29_H_19_N_5_O_5_: C, 67.31; H, 3.70; N, 13.53. Found: C, 67.24; H, 3.79; N, 13.49.

#### N'-(naphthalen-1-ylmethylene)-2,3-diphenylquinoxaline-6-carbohydrazide *7n*

White solid. Yield: 77%. Mp > 250 °C; IR (KBr): υ (cm^−1^) = 3196, 3059, 1695, 1558. ^1^H NMR (301 MHz, DMSO-*d*_6_) δ 12.30 (s, 1H), 9.24 (s, 1H), 8.92 (d, J = 8.6 Hz, 1H), 8.86 (s, 1H), 8.39 (d, *J* = 8.8 Hz, 1H), 8.21 (d, *J* = 8.8 Hz, 1H), 8.05 – 7.88 (m, 3H), 7.67 (t, *J* = 7.7 Hz, 1H), 7.57 (t, *J* = 7.7 Hz, 2H), 7.52 – 7.41 (m, 5H), 7.40 – 7.29 (m, 5H). ^13^C NMR (76 MHz, DMSO-*d*_6_) δ 162.27, 154.76, 154.33, 148.74, 142.28, 140.21, 138.95, 138.92, 134.60, 133.99, 131.11, 130.76, 130.27, 130.19, 129.96, 129.60, 129.45, 129.25, 128.53, 128.27, 127.76, 126.69, 125.96, 124.68 ppm. Anal. calcd. For C_32_H_22_N_4_O: C, 80.32; H, 4.63; N, 11.71. Found: C, 80.25; H, 4.76; N, 11.58.

#### 2,3-diphenyl-N'-(thiophen-2-ylmethylene)quinoxaline-6-carbohydrazide *7o*

White solid. Yield: 84%. Mp 242–246 °C; IR (KBr): υ (cm^−1^) = 3228, 3053, 1715, 1588. ^1^H NMR (301 MHz, DMSO-*d*_6_) δ 12.20 (s, 1H), 8.79 (s, 1H), 8.77 (s, 1H), 8.31 (d, *J* = 8.8 Hz, 1H), 8.20 (d, *J* = 8.7 Hz, 1H), 7.70 (d, *J* = 5.0 Hz, 1H), 7.54 – 7.44 (m, 5H), 7.43 – 7.30 (m, 6H), 7.15 (t, *J* = 4.4 Hz, 1H). ^13^C NMR (76 MHz, DMSO-*d*_6_) δ 162.26, 154.78, 154.37, 143.98, 142.25, 140.17, 139.54, 138.93, 138.90, 134.66, 131.66, 130.25, 130.17, 129.58, 129.45, 129.33, 128.54, 128.34 ppm. Anal. calcd. For C_26_H_18_N_4_OS: C, 71.87; H, 4.18; N, 12.89. Found: C, 71.81; H, 4.12; N, 12.96.

### α-glucosidase inhibition assay

The α-glucosidase inhibitory effects of diphenylquinoxaline-6-carbohydrazide hybrids 7a–o were determined by previously reported method [51]. In this protocol, 20 μL of enzyme solution (α-glucosidase from Saccharomyces cerevisiae, EC3.2.1.20, 20 U/mg), 20 μL of test compounds 7a–o with various concentrations, and 135 μL of potassium phosphate buffer were added and incubated in the 96-well plate for 10 min at 37 ^◦^C. Later on, 25 μL of the substrate (p-nitrophenyl glucopyranoside, 4 mM) was added to each well of the plate, and incubation was continued for 20 min at 37 ^◦^C. Next, absorbance was measured at 405 nm by spectrophotometer (Gen5, Power wave xs2, BioTek, USA), and the IC_50_ value for each tested compound was calculated by taking advantage of the nonlinear regression curve [52, 53].

### Enzyme kinetic studies

The mode of inhibition of the most active compound (**7e**), identified with the lowest IC_50_, was investigated against an α-glucosidase activity with different concentrations of *p*-nitrophenyl *α* -D-glucopyranoside (2–10 mM) as substrate in the absence and presence of sample **7e** at different concentrations (0, 30, 70 and 110 µM). A Lineweaver–Burk plot was generated to identify the type of inhibition and the Michaelis–Menten constant (*K*_m_) value was determined from the plot between reciprocal of the substrate concentration (1/[S]) and reciprocal of enzyme rate (1/V) over various inhibitor concentrations. The experimental inhibitor constant (*K*_i_) value was constructed by secondary plots of the inhibitor concentration [I] versus *K*_m_ [43, 54].

### Molecular docking

The molecular docking investigation of all derivatives were performed using the maestro molecular modeling platform (version 10.5), Schrödinger suites [55]. X-ray crystallographic structure of α-glucosidase 5NN8 was downloaded from the PDB website (https://www.rcsb.org/) [54]. A protein preparation wizard was used to remove water molecules and co-crystallized atoms from the protein and prepare the receptor. Moreover, heteroatom states were generated at pH: 7.4 by EPIK, and H-bonds were assigned using PROPKA at the same pH. 2D structure of ligands was drawn in Hyperchem, energy minimized using, molecular mechanics and molecular quantum approaches. Next, the ligand preparation wizard was used to prepare the ligand using the OPLS_2005 force field [56]. Acarbose all compounds were docked into the binding sites using glide tasked to report five poses per ligand with flexible ligand sampling and extra precision [17, 43].

## Supplementary Information

**Additional file 1: Fig. S1**. (E)-N'-benzylidene-2,3-diphenylquinoxaline-6-carbohydrazide (7a). **Fig. S2**. (E)-N'-(2-nitrobenzylidene)-2,3-diphenylquinoxaline-6-carbohydrazide (7b). **Fig. S3**. (E)-N'-(3-nitrobenzylidene)-2,3-diphenylquinoxaline-6-carbohydrazide (7c). **Fig. S4**. (E)-N'-(4-nitrobenzylidene)-2,3-diphenylquinoxaline-6-carbohydrazide (7d). **Fig. S5**. (E)-N'-(3-fluorobenzylidene)-2,3-diphenylquinoxaline-6-carbohydrazide (7e). **Fig. S6**. (E)-N'-(4-chlorobenzylidene)-2,3-diphenylquinoxaline-6-carbohydrazide (7f). **Fig. S7**. (E)-N'-(4-methoxybenzylidene)-2,3-diphenylquinoxaline-6-carbohydrazide (7g). **Fig. S8**. (E)-N'-(3-methoxy-2-nitrobenzylidene)-2,3-diphenylquinoxaline-6-carbohydrazide (7h). **Fig. S9**. (E)-N'-(2-chloro-5-nitrobenzylidene)-2,3-diphenylquinoxaline-6-carbohydrazide (7i). **Fig. S10**. (E)-N'-(4-hydroxy-3-methoxybenzylidene)-2,3-diphenylquinoxaline-6-carbohydrazide (7j). **Fig. S11**. (E)-2,3-diphenyl-N'-(3,4,5-trimethoxybenzylidene)quinoxaline-6-carbohydrazide (7k). **Fig. S12**. (E)-N'-(3-phenoxybenzylidene)-2,3-diphenylquinoxaline-6-carbohydrazide (7l). **Fig. S13**. (E)-N'-((6-nitrobenzo[d][1,3]dioxol-5-yl)methylene)-2,3-diphenylquinoxaline-6-carbohydrazide (7m). **Fig. S14**. (E)-N'-(naphthalen-1-ylmethylene)-2,3-diphenylquinoxaline-6-carbohydrazide (7n). **Fig. S15**. (E)-2,3-diphenyl-N'-(thiophen-2-ylmethylene)quinoxaline-6-carbohydrazide (7o).

## Data Availability

The datasets generated and/or analysed during the current study are available in the Worldwide Protein Data Bank (wwPDB) repository. (http://www.rcsb.org).
