# Supplementary material for: Design, synthesis, and molecular docking studies of diphenylquinoxaline-6-carbohydrazide hybrids as potent α-glucosidase inhibitors
Source: BMC Chem. 2022 Jul 31;16(1):57. doi: 10.1186/s13065-022-00848-4 (PMC9341091; doi:10.1186/s13065-022-00848-4)
Supplement: Supplementary file 1 — Additional file 1: Fig. S1. (E)-N'-benzylidene-2,3-diphenylquinoxaline-6-carbohydrazide (7a). Fig. S2. (E)-N'-(2-nitrobenzylidene)-2,3-diphenylquinoxaline-6-carbohydrazide (7b). Fig. S3. (E)-N'-(3-nitrobenzylidene)-2,3-diphenylquinoxaline-6-carbohydrazide (7c). Fig. S4. (E)-N'-(4-nitrobenzylidene)-2,3-diphenylquinoxaline-6-carbohydrazide (7d). Fig. S5. (E)-N'-(3-fluorobenzylidene)-2,3-diphenylquinoxaline-6-carbohydrazide (7e). Fig. S6. (E)-N'-(4-chlorobenzylidene)-2,3-diphenylquinoxaline-6-carbohydrazide (7f). Fig. S7. (E)-N'-(4-methoxybenzylidene)-2,3-diphenylquinoxaline-6-carbohydrazide (7g). Fig. S8. (E)-N'-(3-methoxy-2-nitrobenzylidene)-2,3-diphenylquinoxaline-6-carbohydrazide (7h). Fig. S9. (E)-N'-(2-chloro-5-nitrobenzylidene)-2,3-diphenylquinoxaline-6-carbohydrazide (7i). Fig. S10. (E)-N'-(4-hydroxy-3-methoxybenzylidene)-2,3-diphenylquinoxaline-6-carbohydrazide (7j). Fig. S11. (E)-2,3-diphenyl-N'-(3,4,5-trimethoxybenzylidene)quinoxaline-6-carbohydrazide (7k). Fig. S12. (E)-N'-(3-phenoxybenzylidene)-2,3-diphenylquinoxaline-6-carbohydrazide (7l). Fig. S13. (E)-N'-((6-nitrobenzo[d][1,3]dioxol-5-yl)methylene)-2,3-diphenylquinoxaline-6-carbohydrazide (7m). Fig. S14. (E)-N'-(naphthalen-1-ylmethylene)-2,3-diphenylquinoxaline-6-carbohydrazide (7n). Fig. S15. (E)-2,3-diphenyl-N'-(thiophen-2-ylmethylene)quinoxaline-6-carbohydrazide (7o). [file 13065_2022_848_MOESM1_ESM.docx]

**Fig. S1. (E)-N'-benzylidene-2,3-diphenylquinoxaline-6-carbohydrazide (7a)**

**
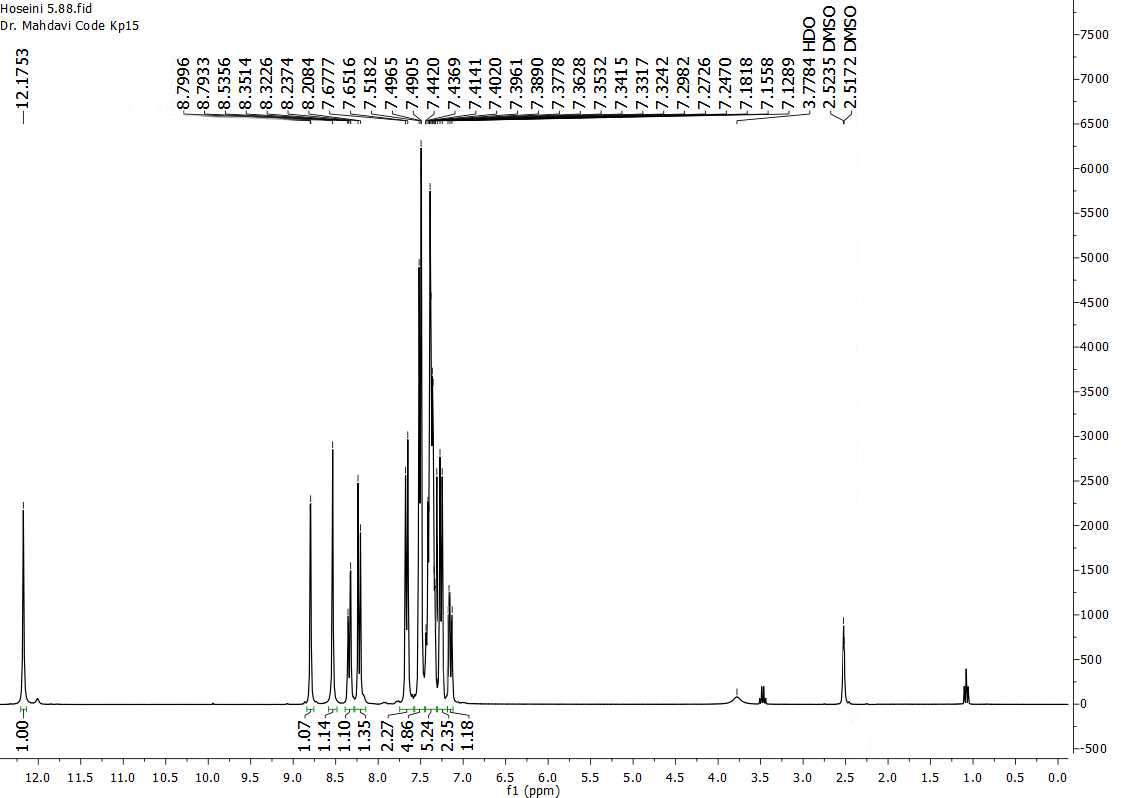
**

**
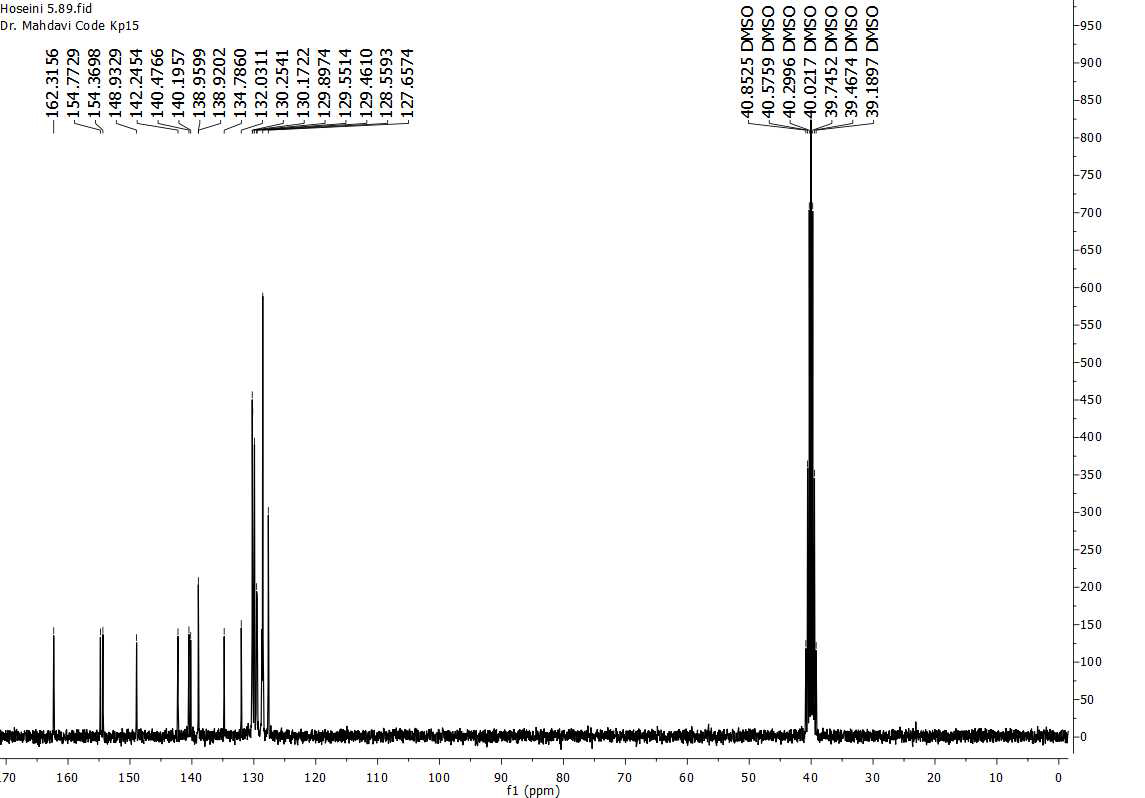
**

**Fig. S2. (E)-N'-(2-nitrobenzylidene)-2,3-diphenylquinoxaline-6-carbohydrazide (7b)**

**
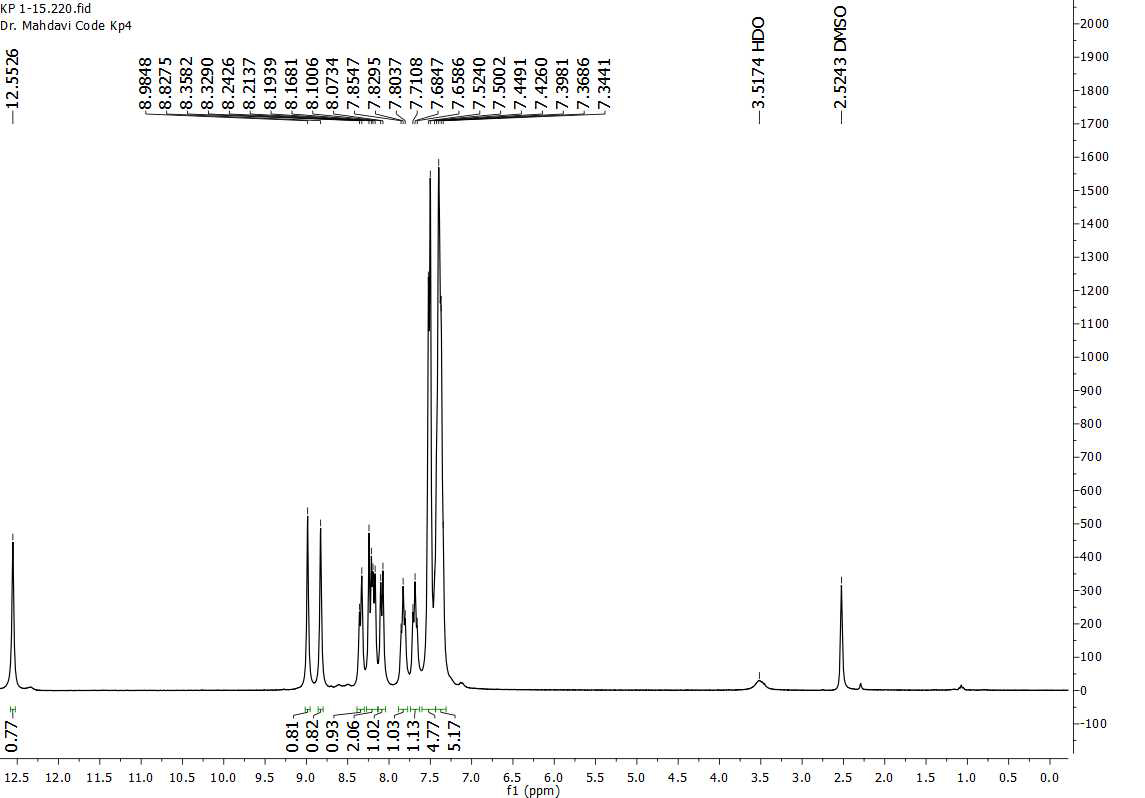
**

**
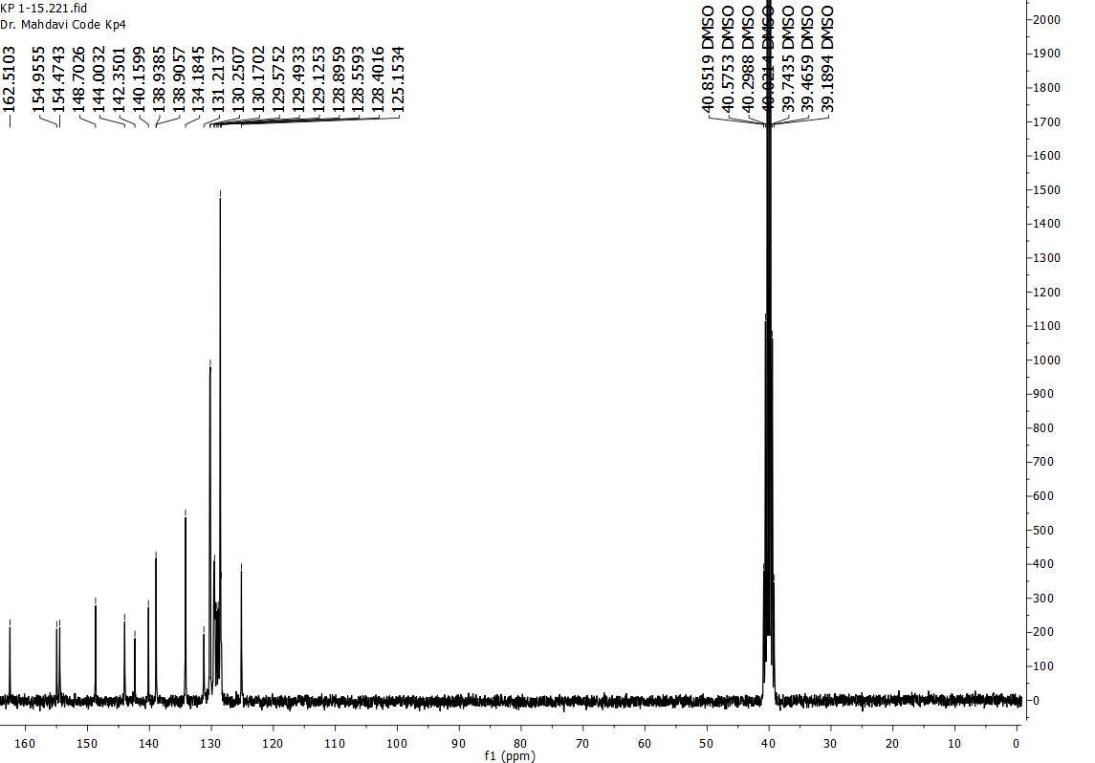
**

**Fig. S3. (E)-N'-(3-nitrobenzylidene)-2,3-diphenylquinoxaline-6-carbohydrazide (7c)**

**
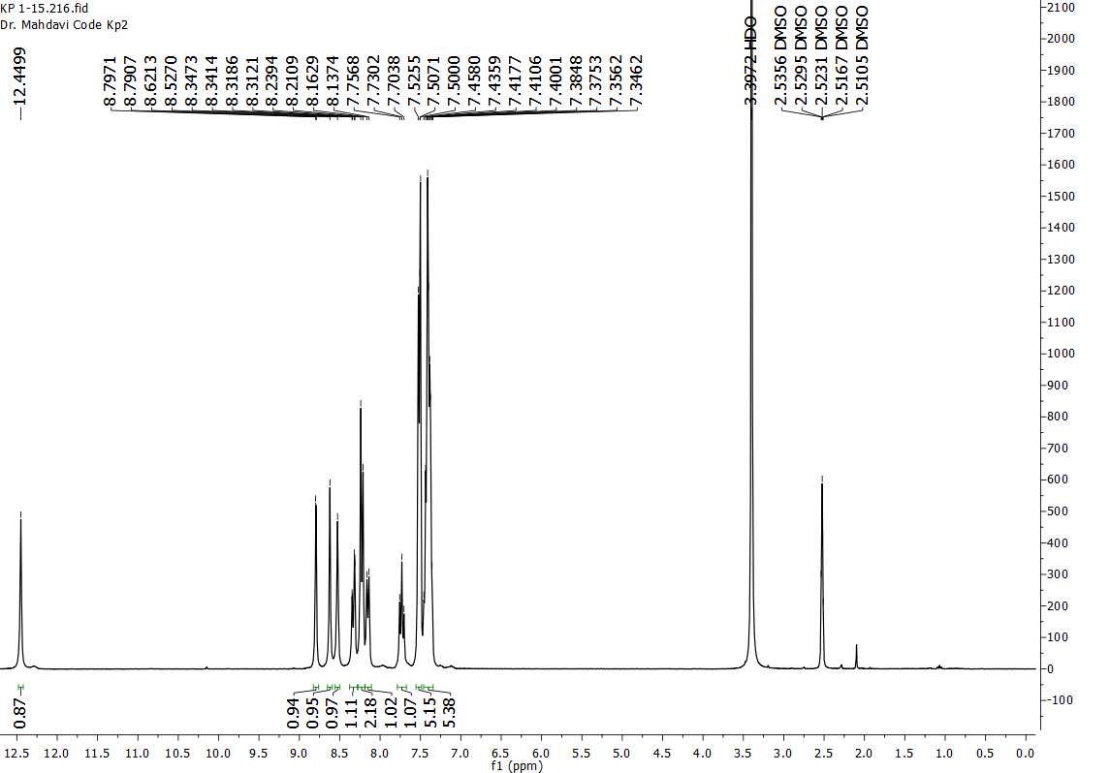
**

**
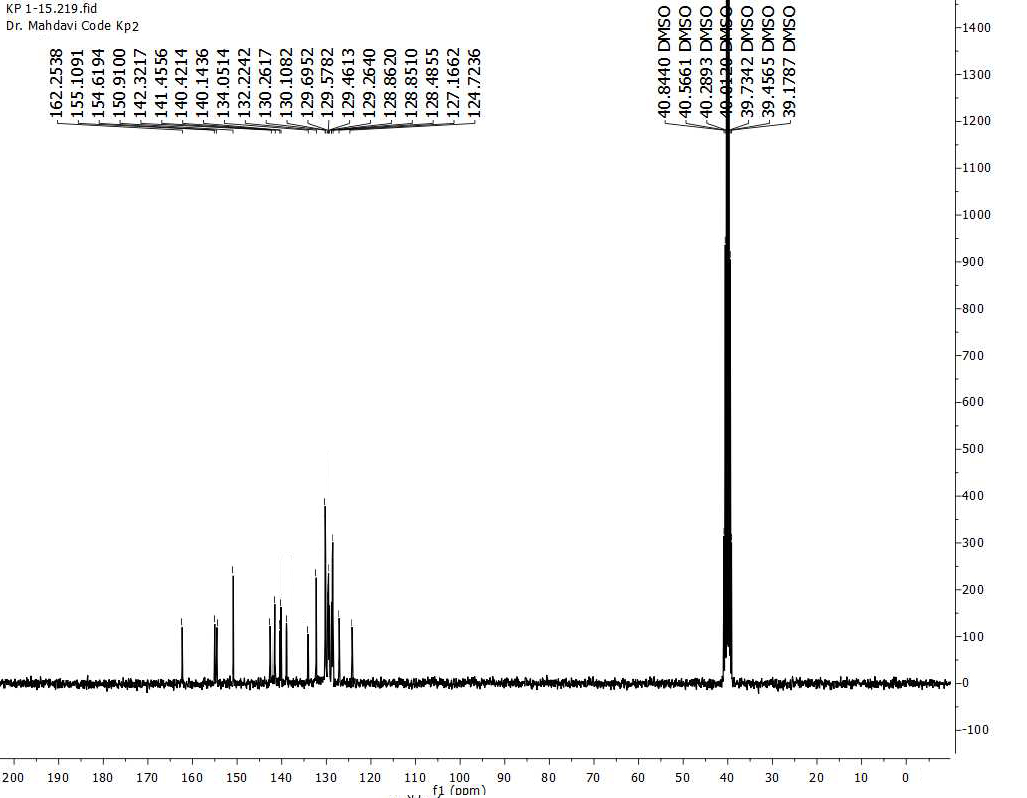
**

**Fig. S4. (E)-N'-(4-nitrobenzylidene)-2,3-diphenylquinoxaline-6-carbohydrazide (7d)**

**
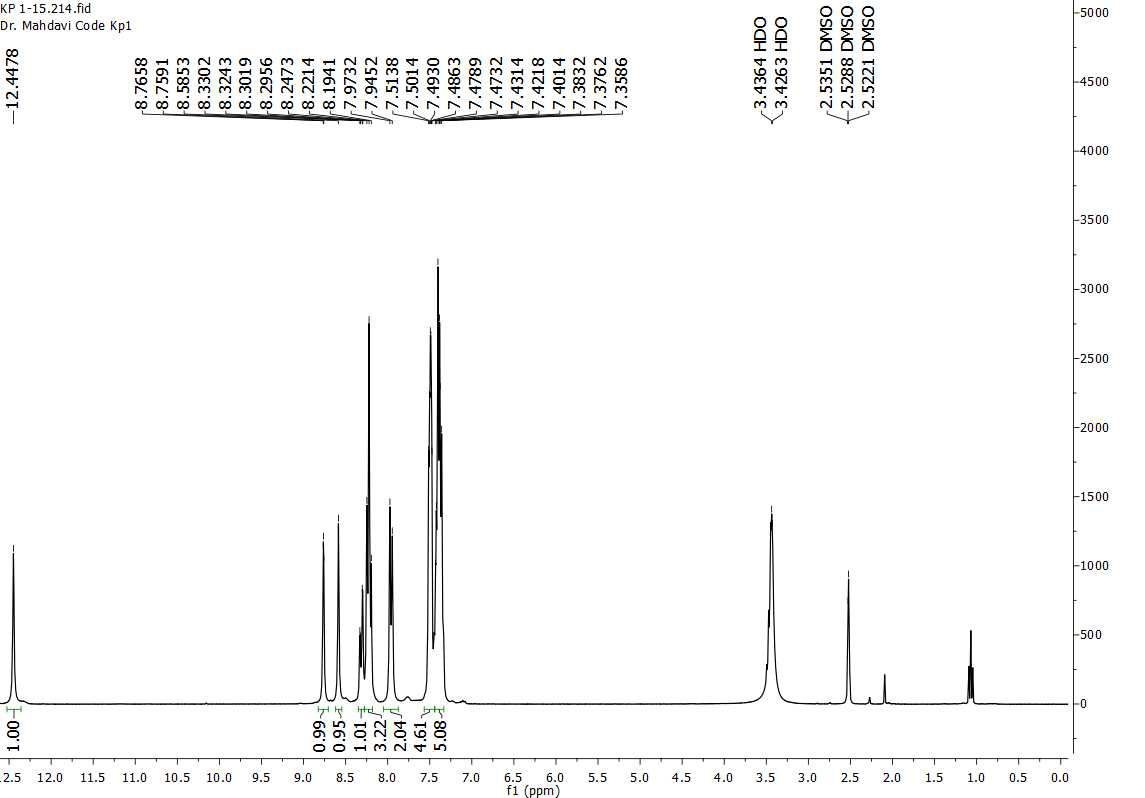

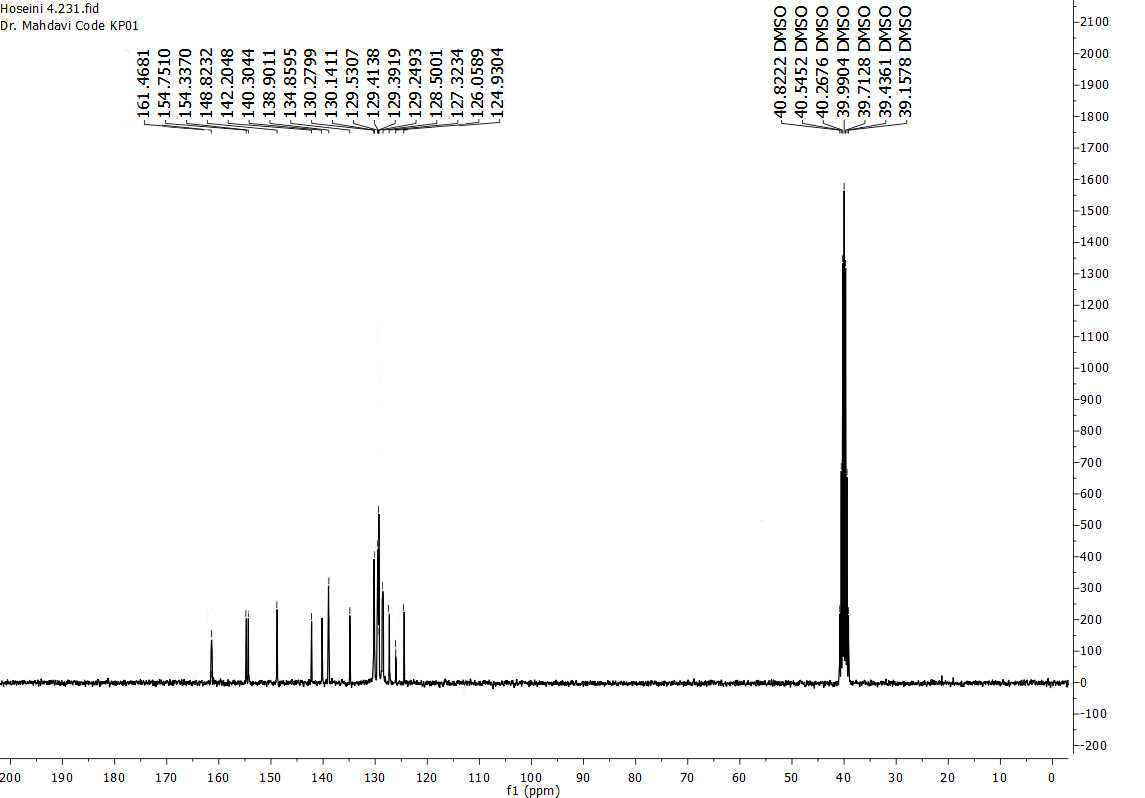
**

**Fig. S5. (E)-N'-(3-fluorobenzylidene)-2,3-diphenylquinoxaline-6-carbohydrazide (7e)**

**
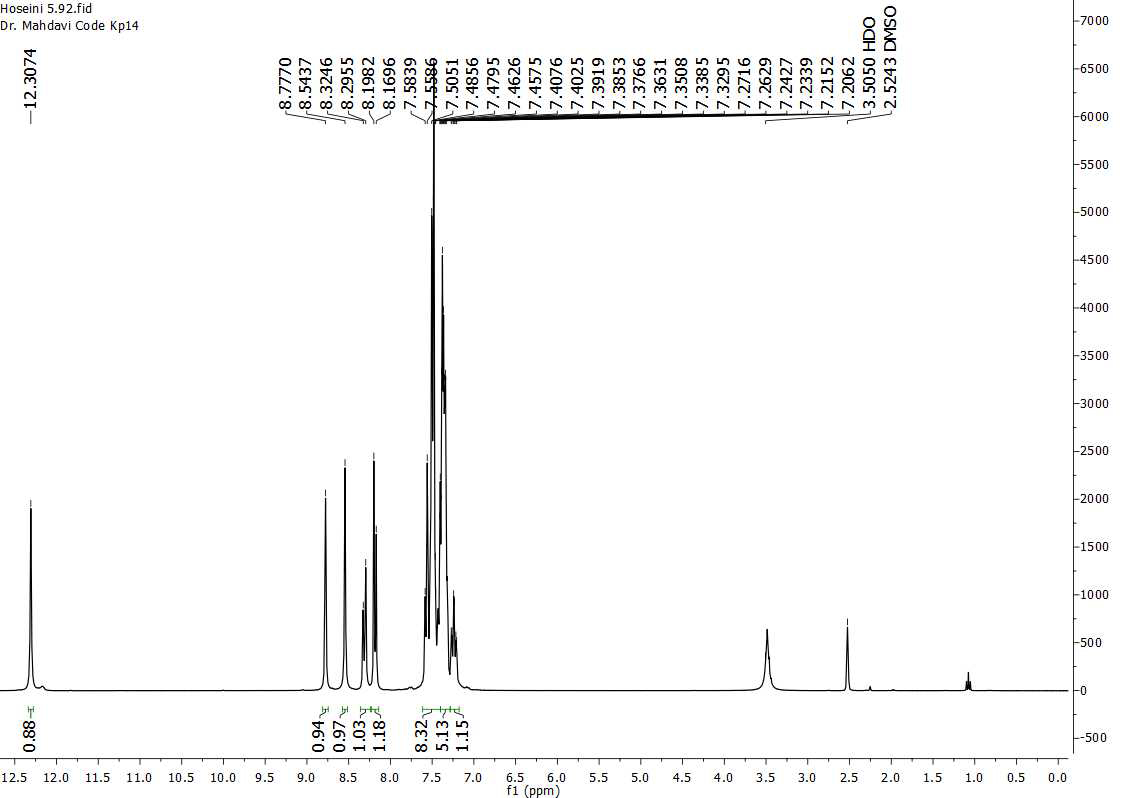
**

**
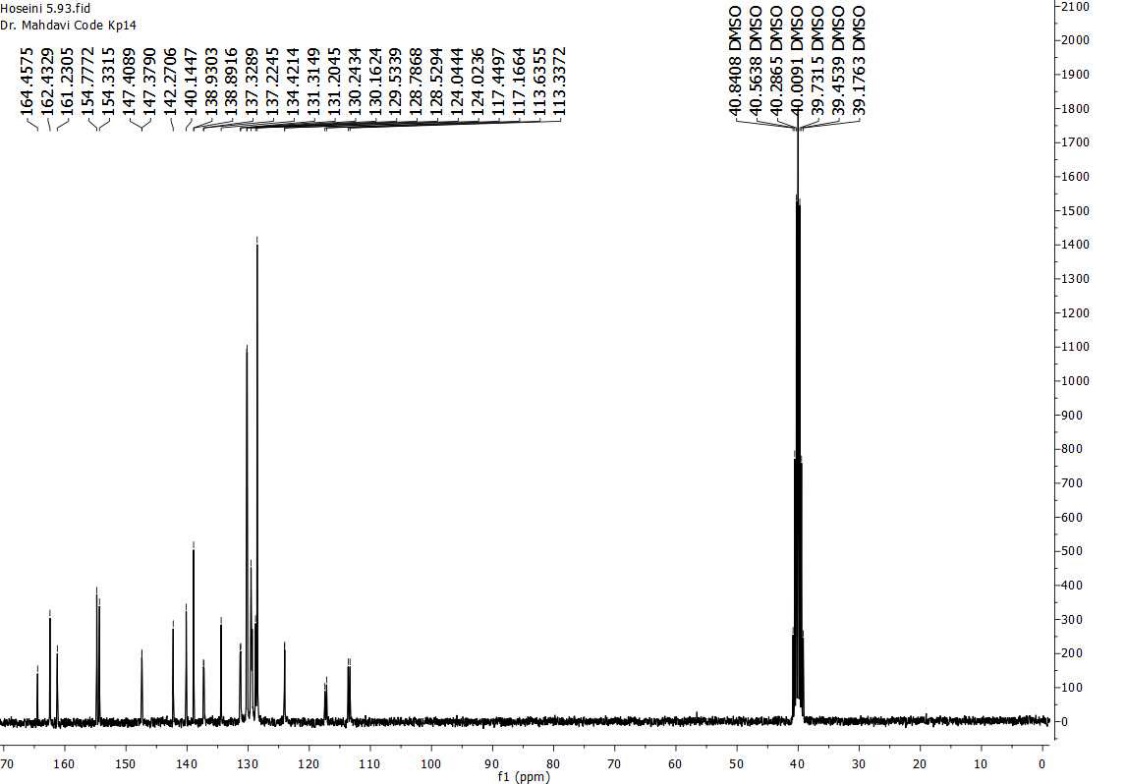
**

**Fig. S6. (E)-N'-(4-chlorobenzylidene)-2,3-diphenylquinoxaline-6-carbohydrazide (7f)**

**
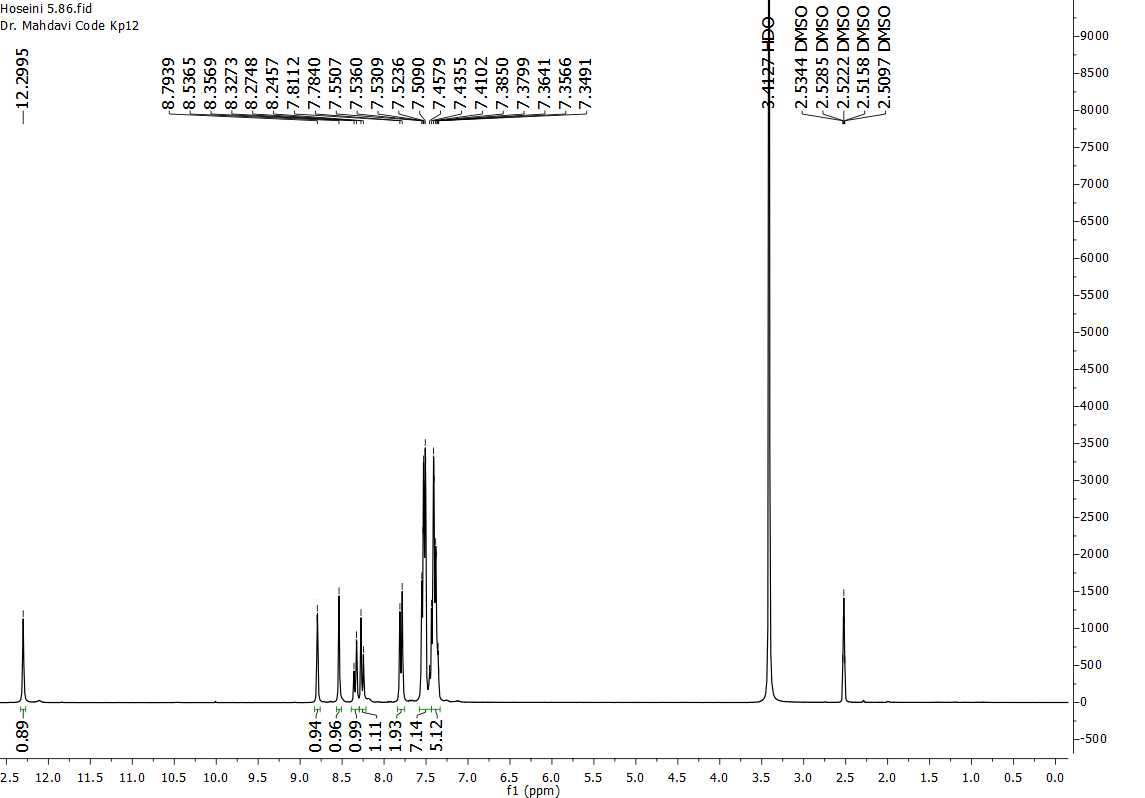
**

**
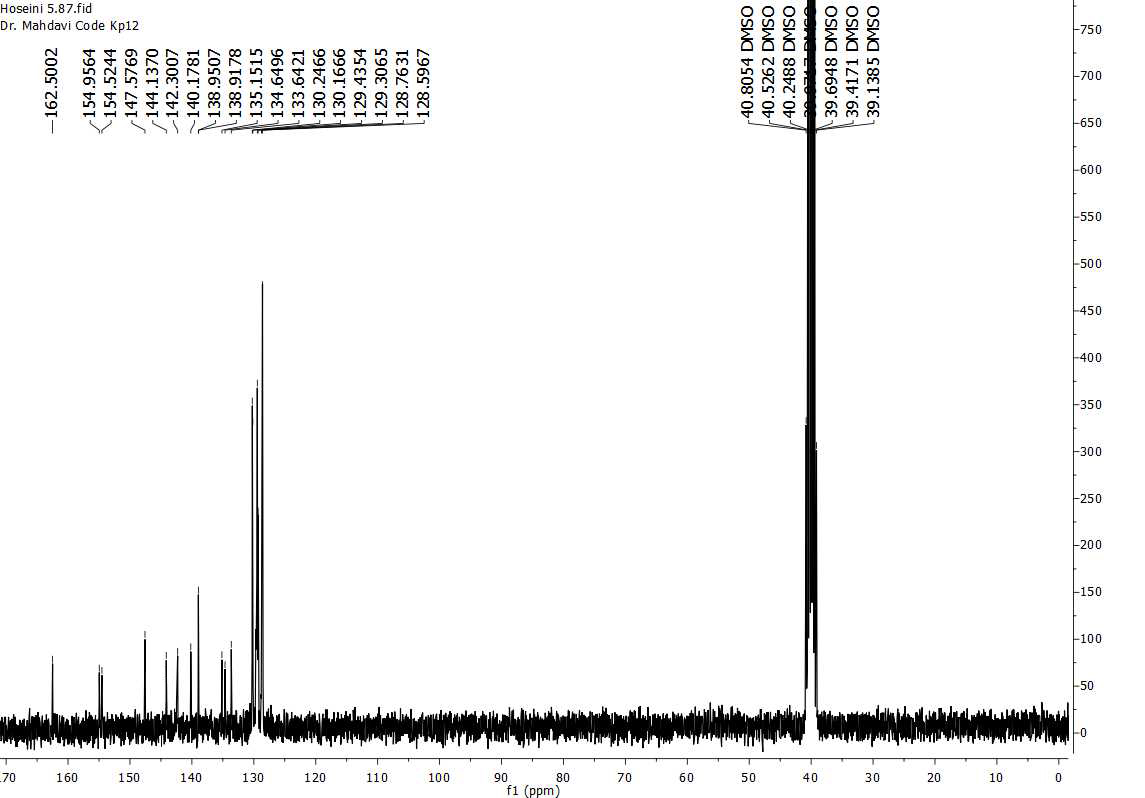
**

**Fig. S7. (E)-N'-(4-methoxybenzylidene)-2,3-diphenylquinoxaline-6-carbohydrazide (7g)**

**
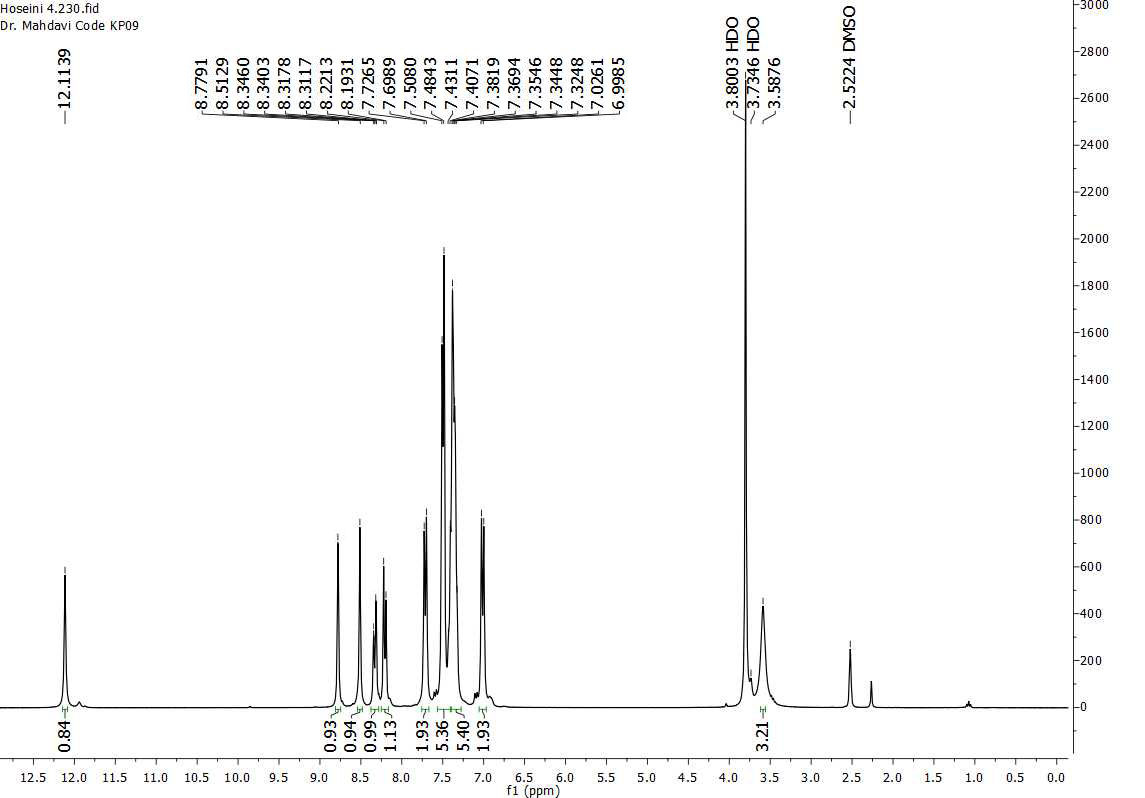
**

**
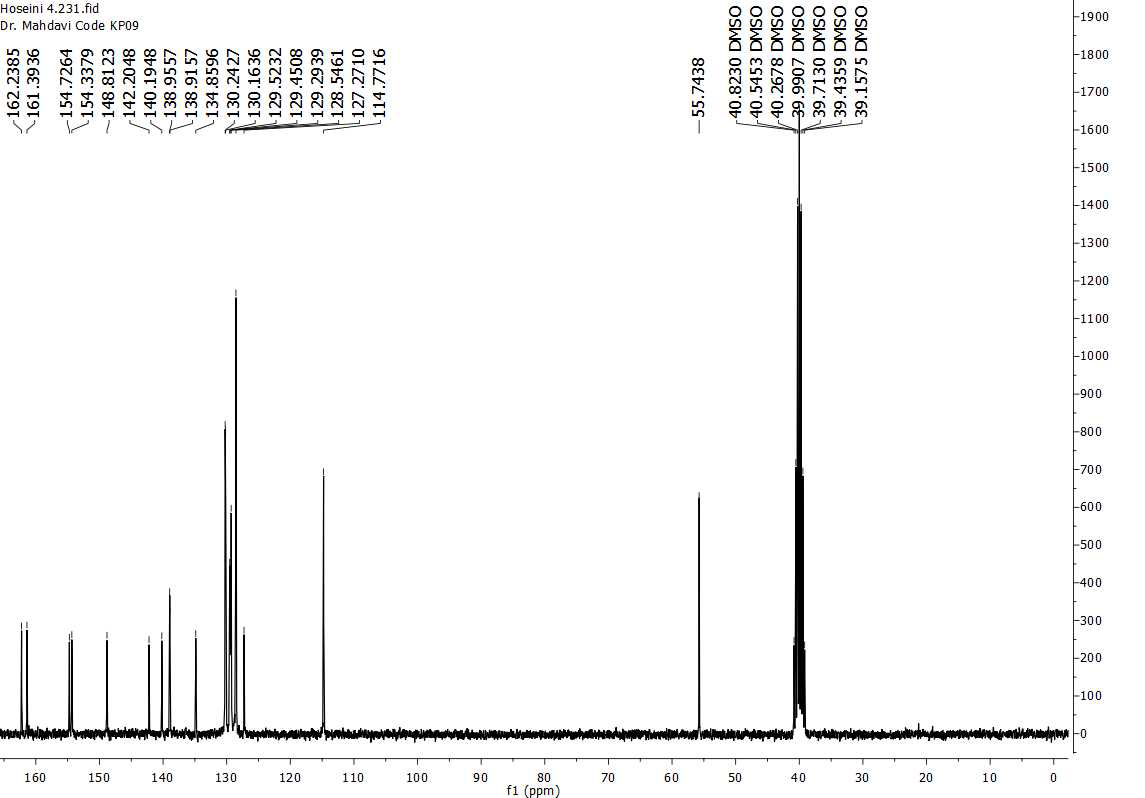
**

**Fig. S8. (E)-N'-(3-methoxy-2-nitrobenzylidene)-2,3-diphenylquinoxaline-6-carbohydrazide (7h)**

**
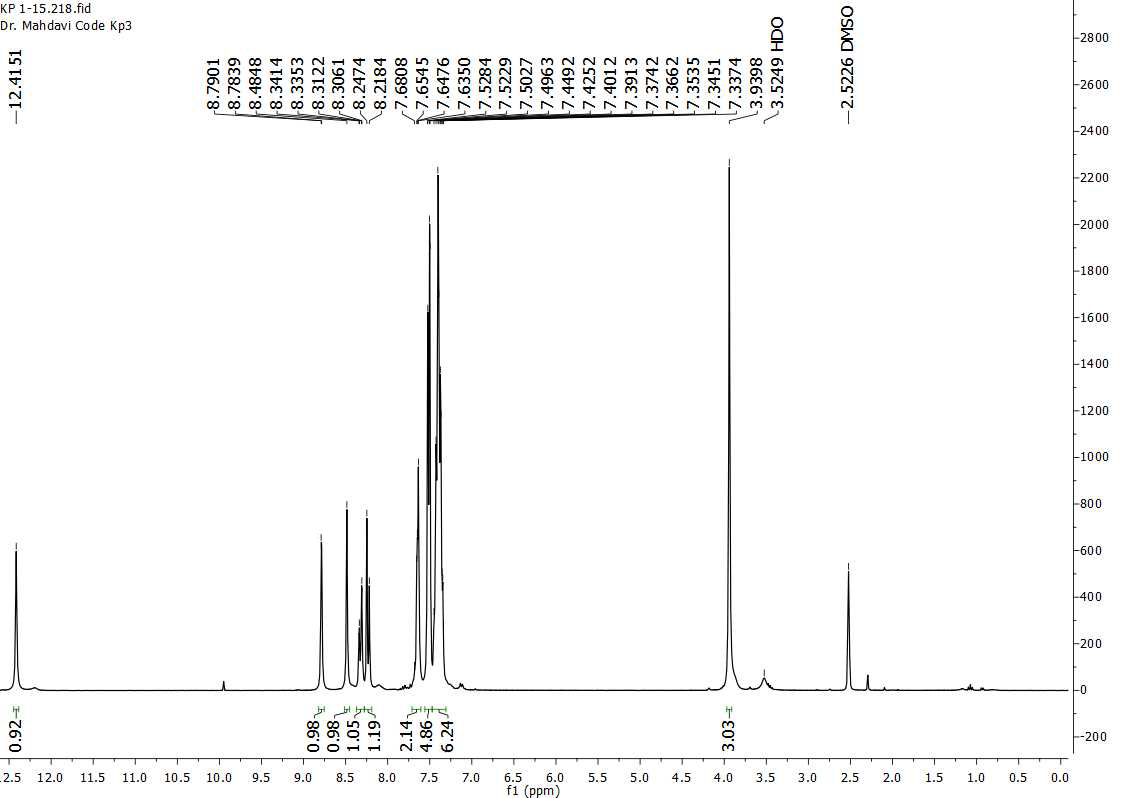
**

**
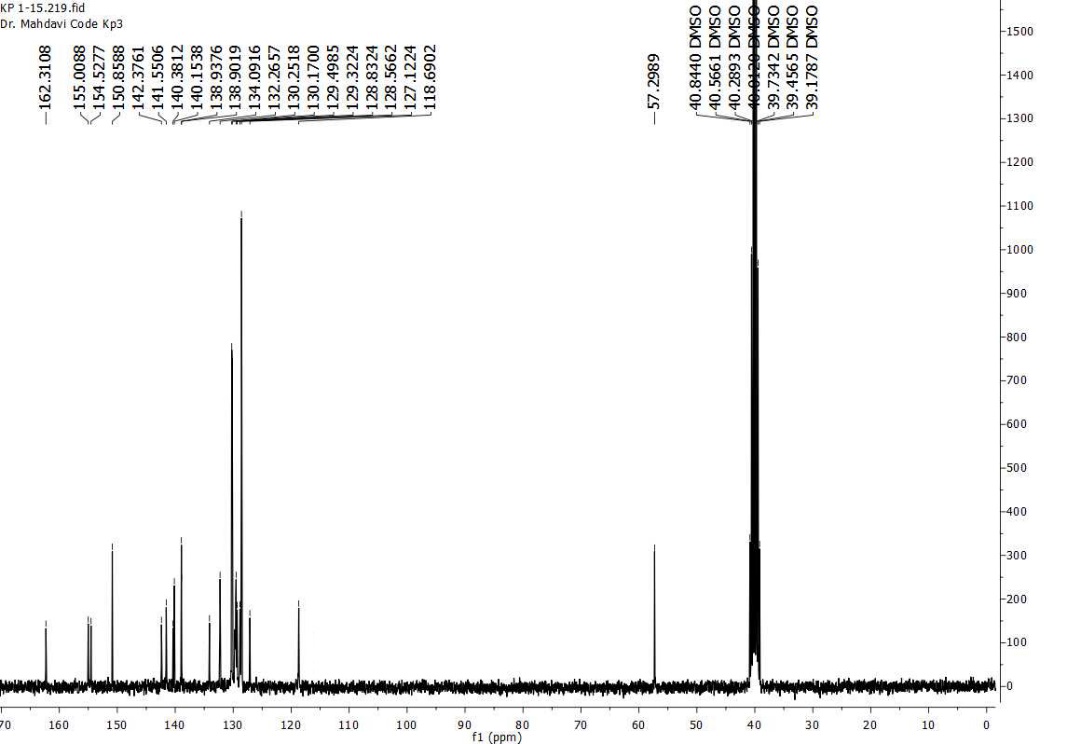
**

**Fig. S9. (E)-N'-(2-chloro-5-nitrobenzylidene)-2,3-diphenylquinoxaline-6-carbohydrazide (7i)**

**
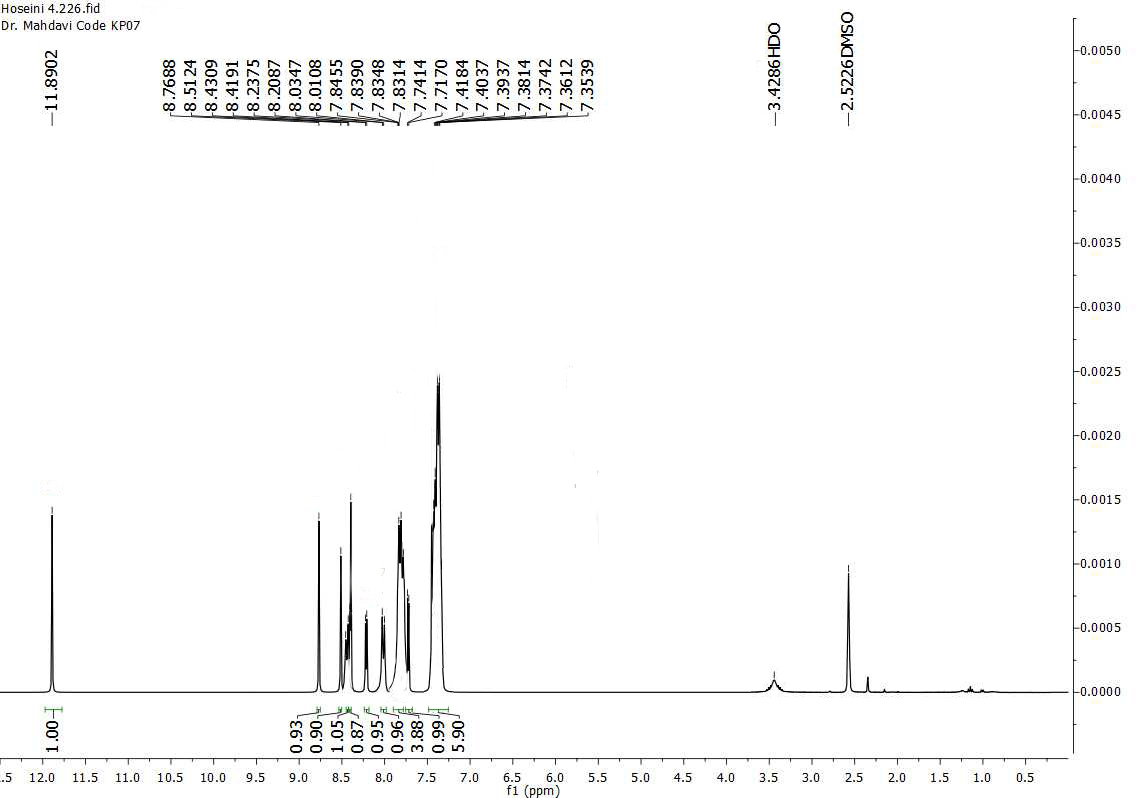
**

**
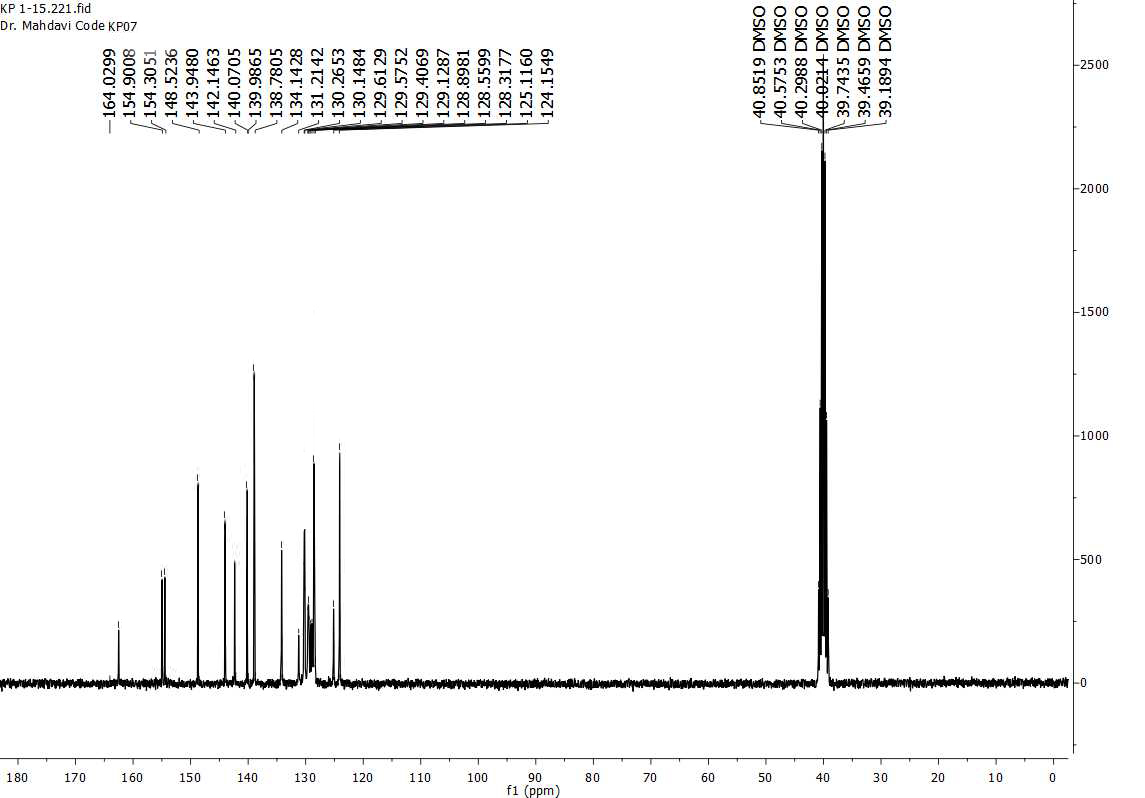
**

**Fig. S10. (E)-N'-(4-hydroxy-3-methoxybenzylidene)-2,3-diphenylquinoxaline-6-carbohydrazide (7j)**

**
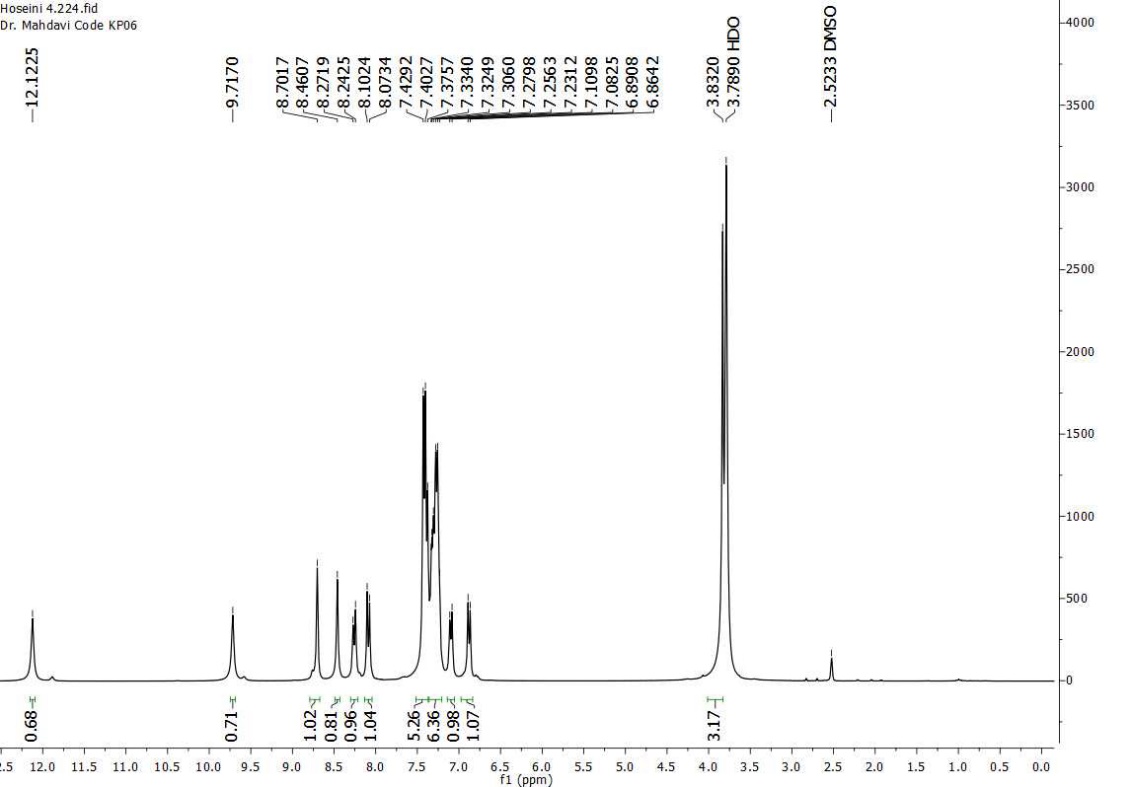
**


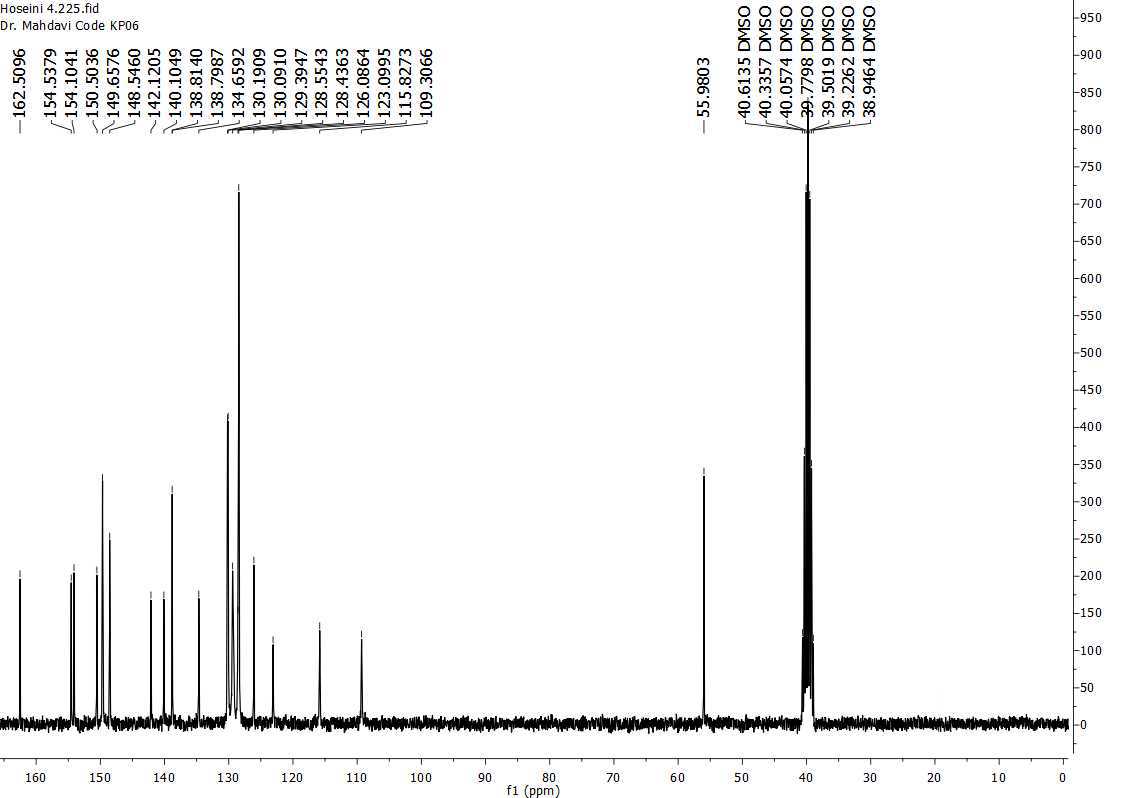

**Fig. S11. (E)-2,3-diphenyl-N'-(3,4,5-trimethoxybenzylidene)quinoxaline-6-carbohydrazide (7k)**

**
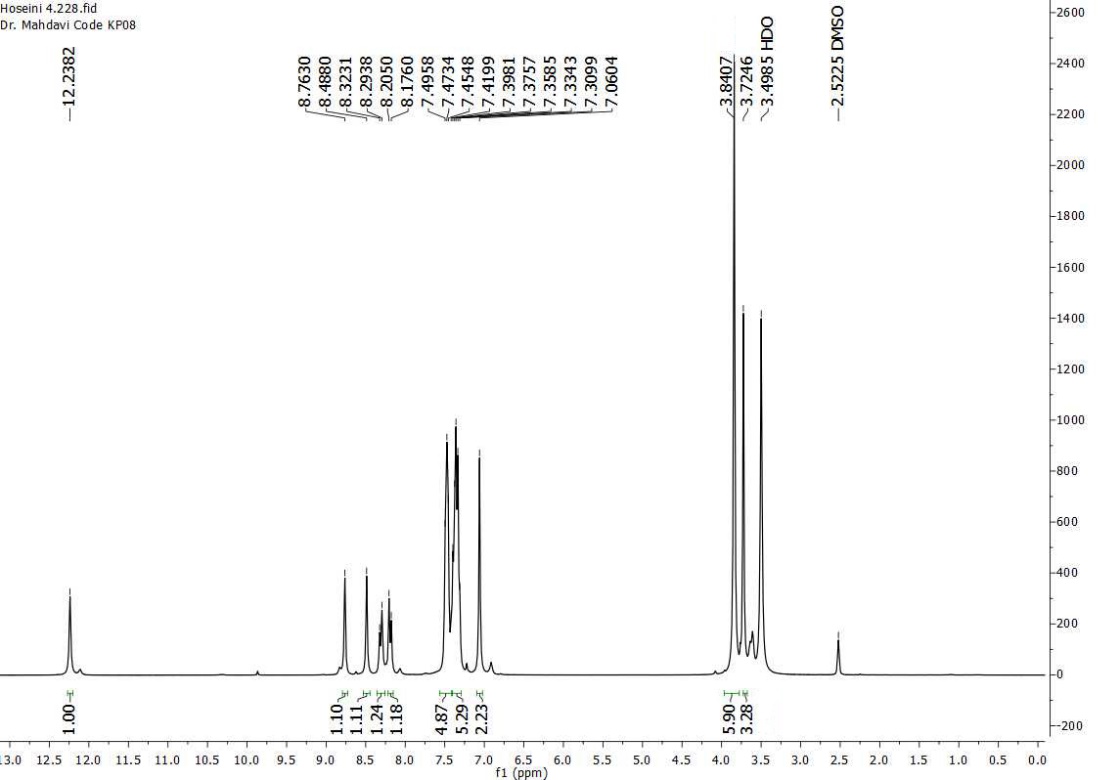
**

**
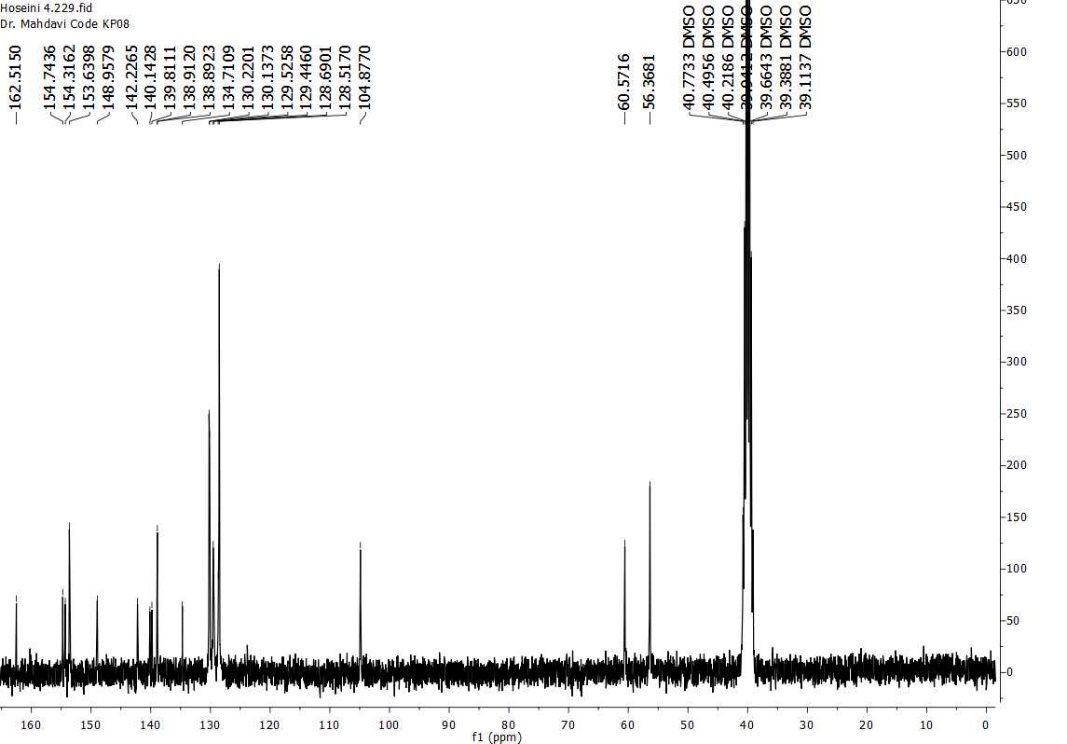
**

**Fig. S12. (E)-N'-(3-phenoxybenzylidene)-2,3-diphenylquinoxaline-6-carbohydrazide (7l)**

**
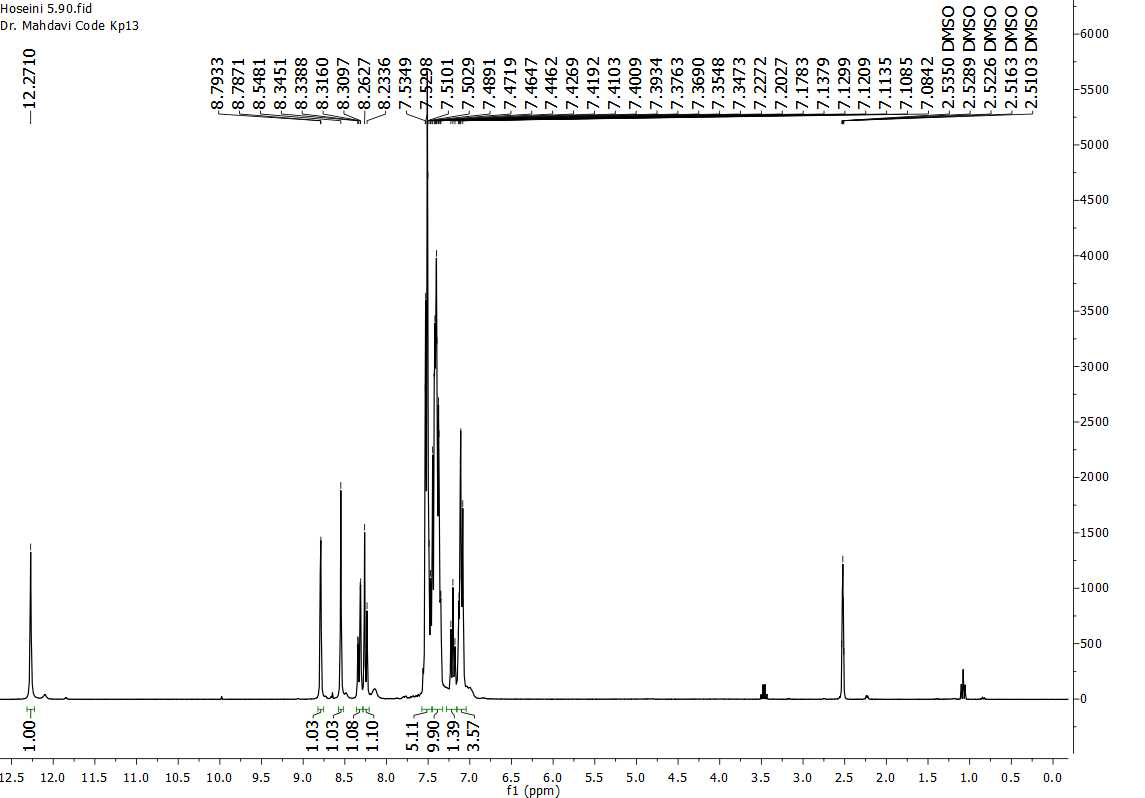
**

**
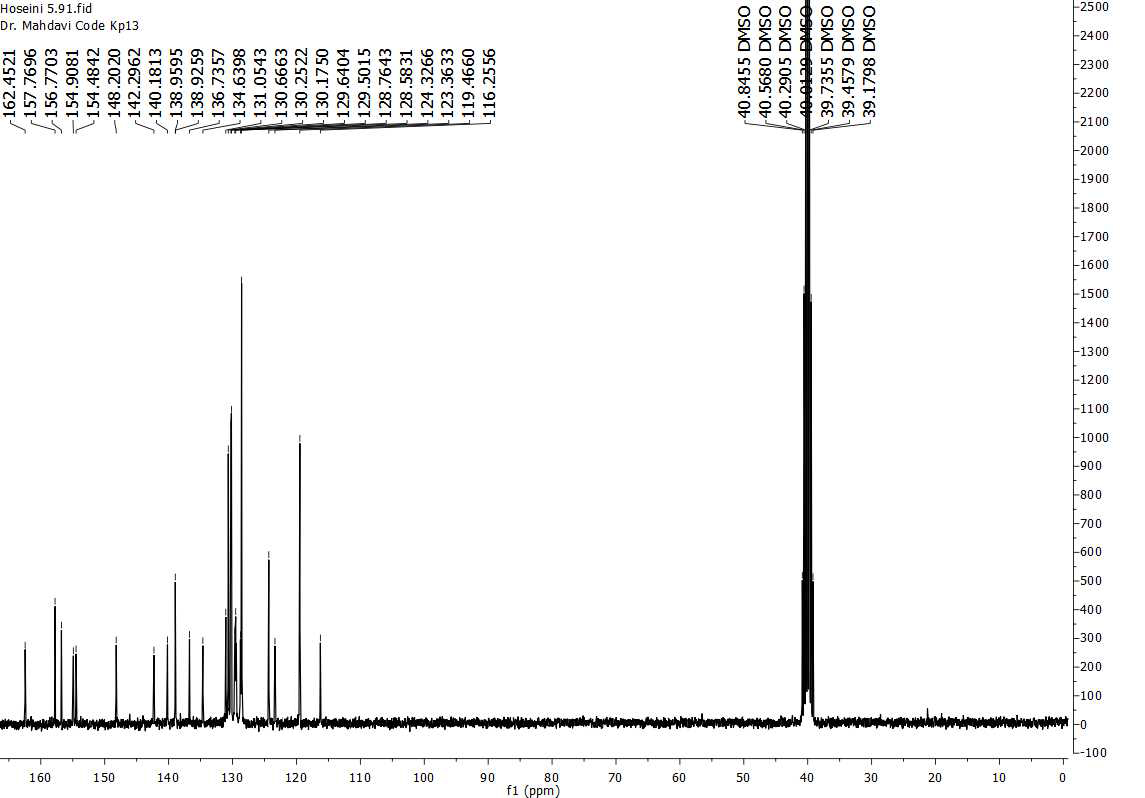
**

**

**Fig. S13. (E)-N'-((6-nitrobenzo[d][1,3]dioxol-5-yl)methylene)-2,3-diphenylquinoxaline-6-carbohydrazide (7m)**


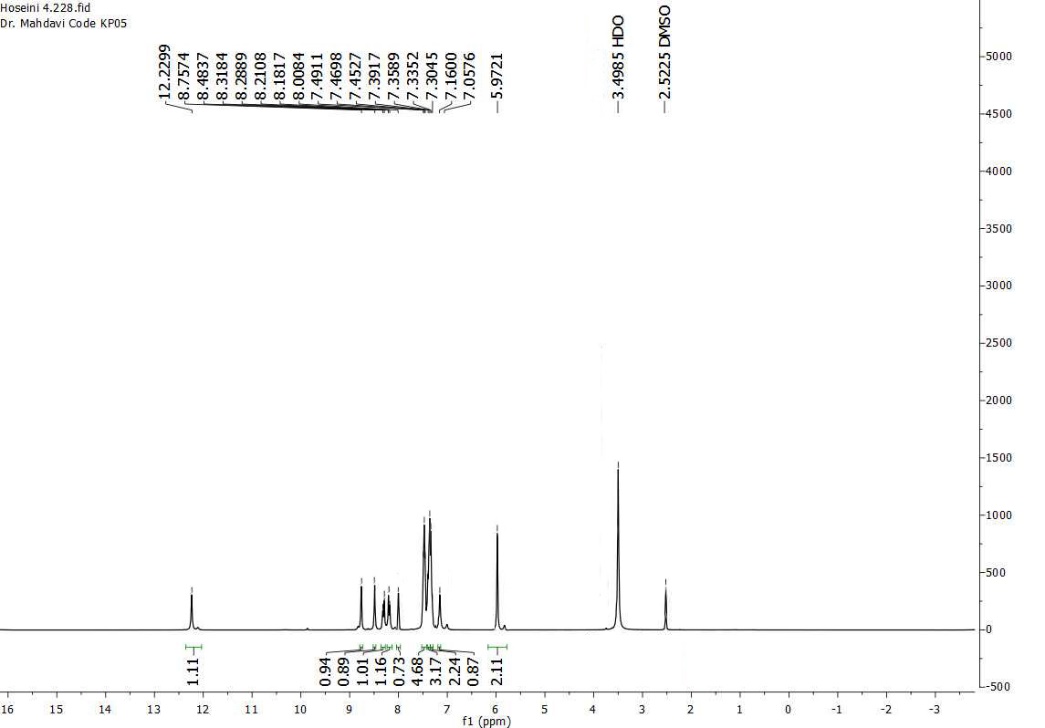


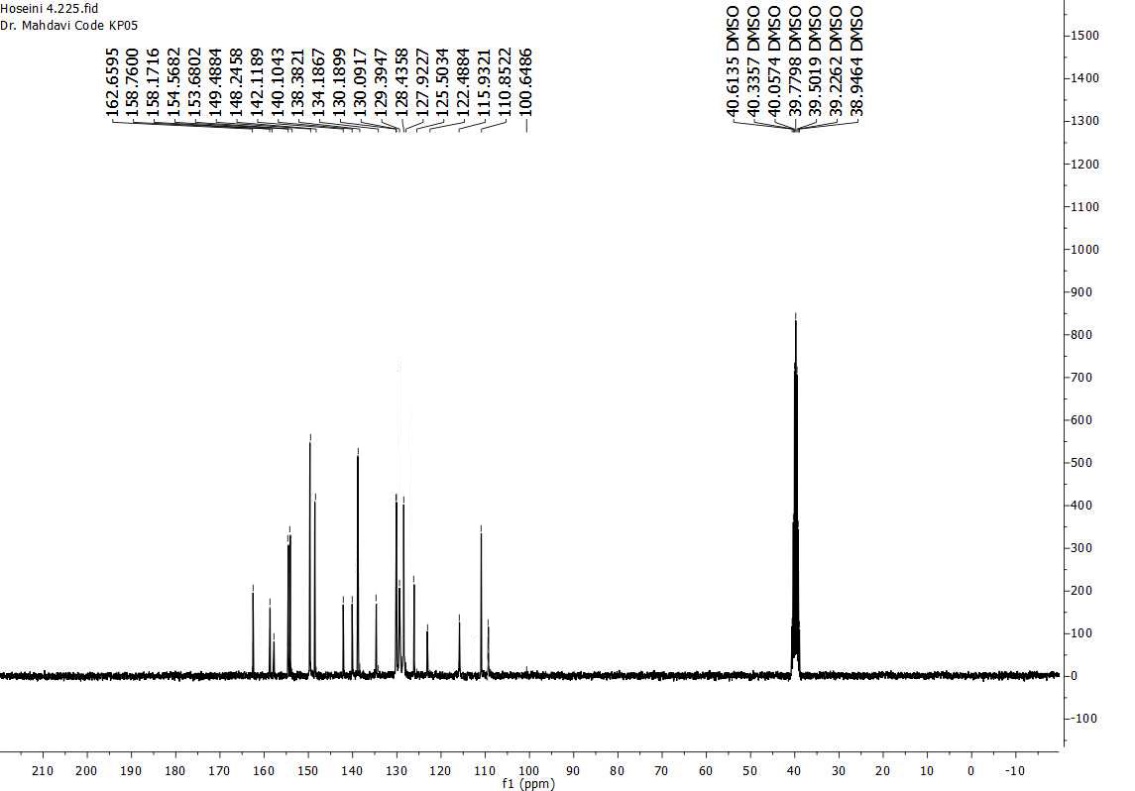

**Fig. S14. (E)-N'-(naphthalen-1-ylmethylene)-2,3-diphenylquinoxaline-6-carbohydrazide (7n)**

**
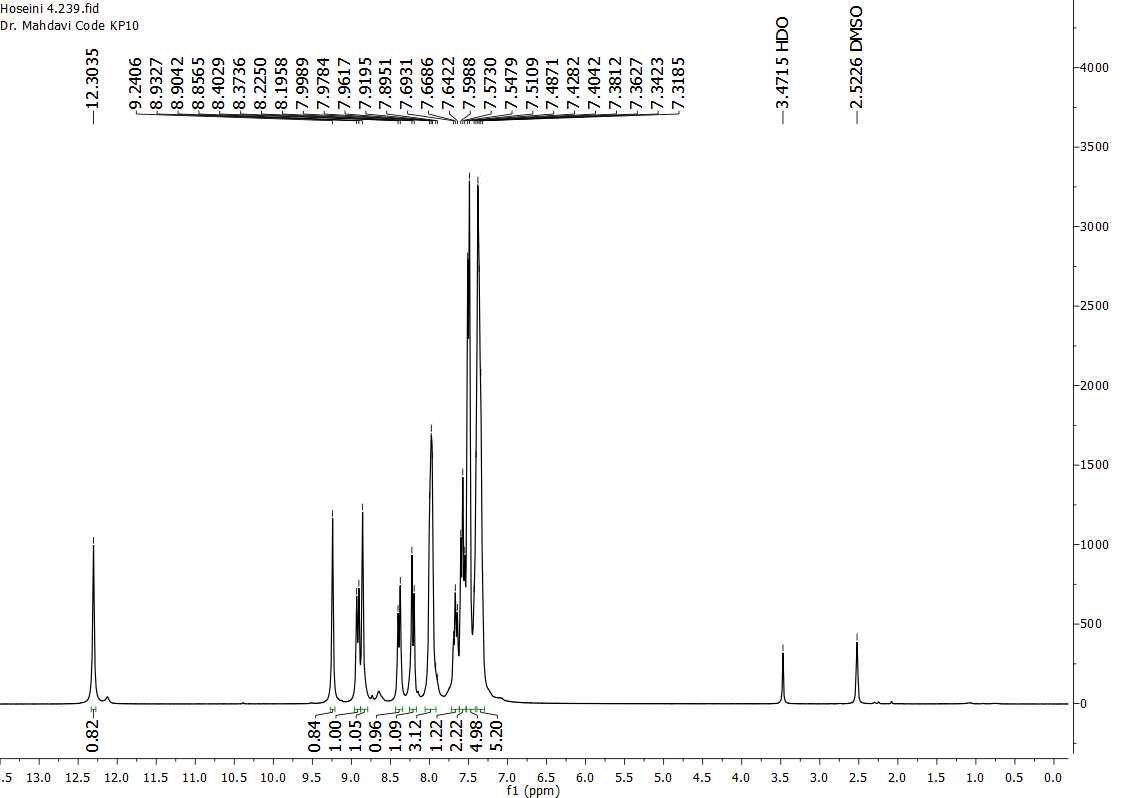
**

**
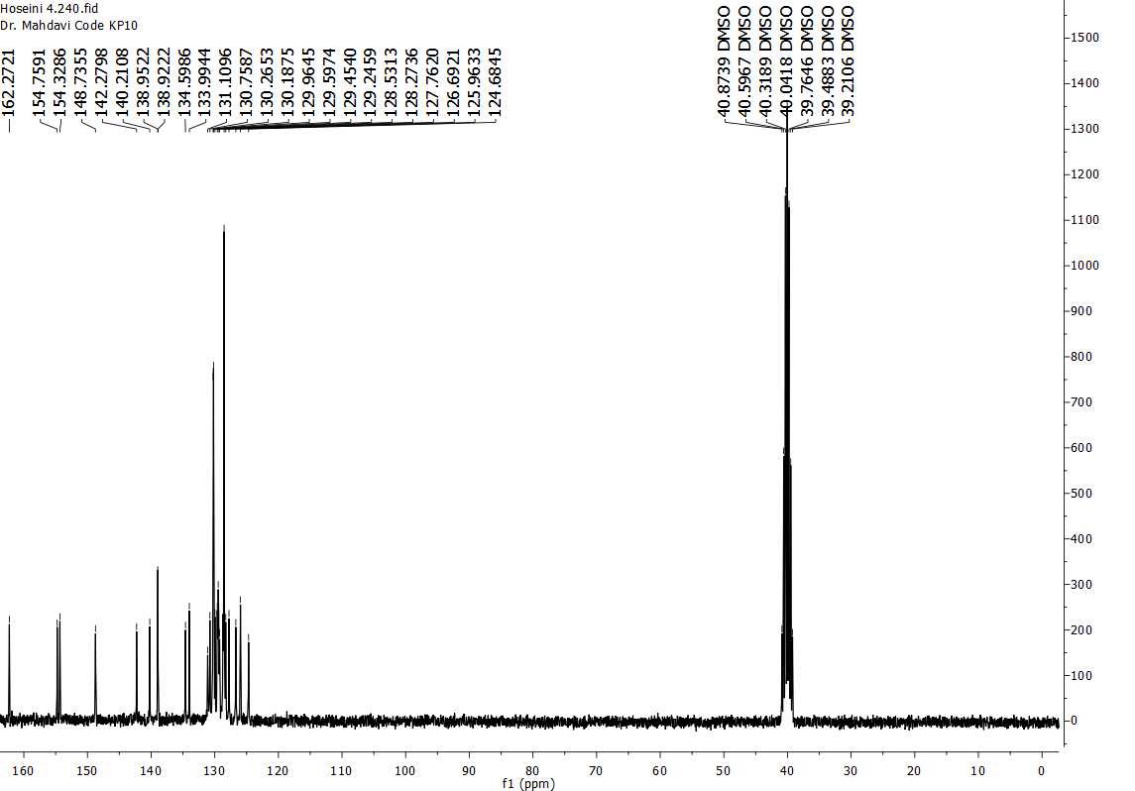
**

**Fig. S15. (E)-2,3-diphenyl-N'-(thiophen-2-ylmethylene)quinoxaline-6-carbohydrazide (7o)**

**
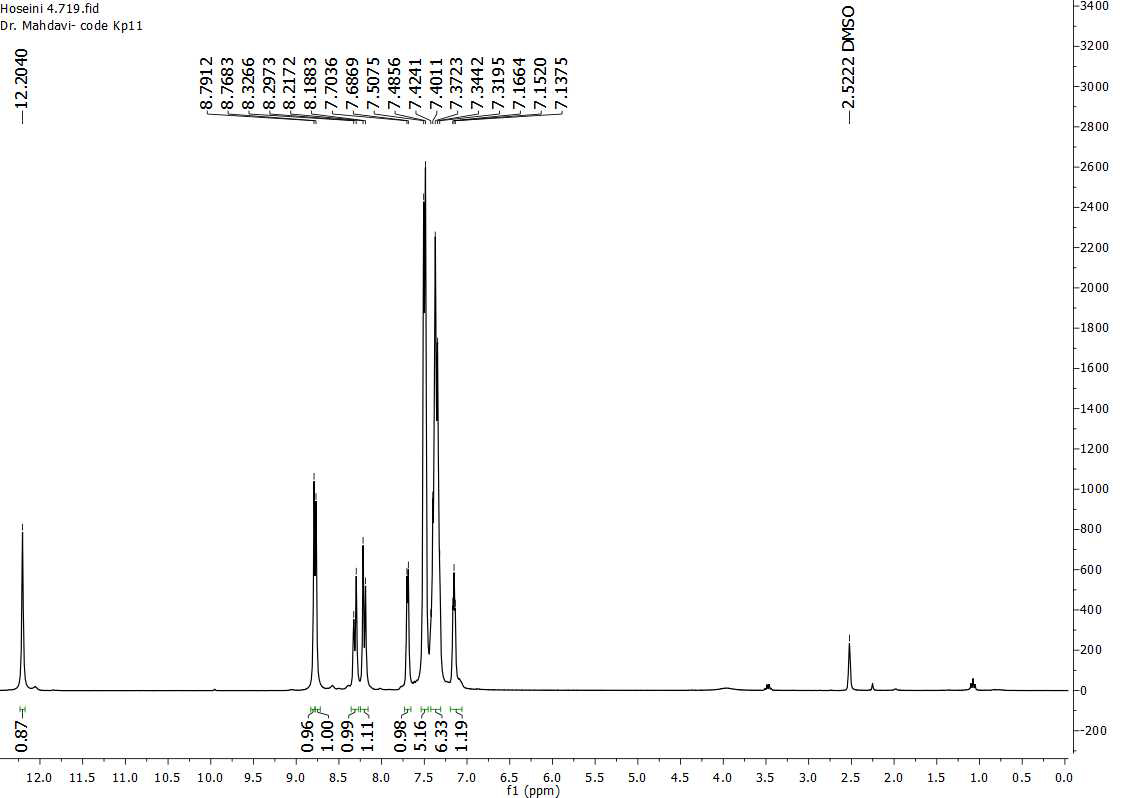
**

**
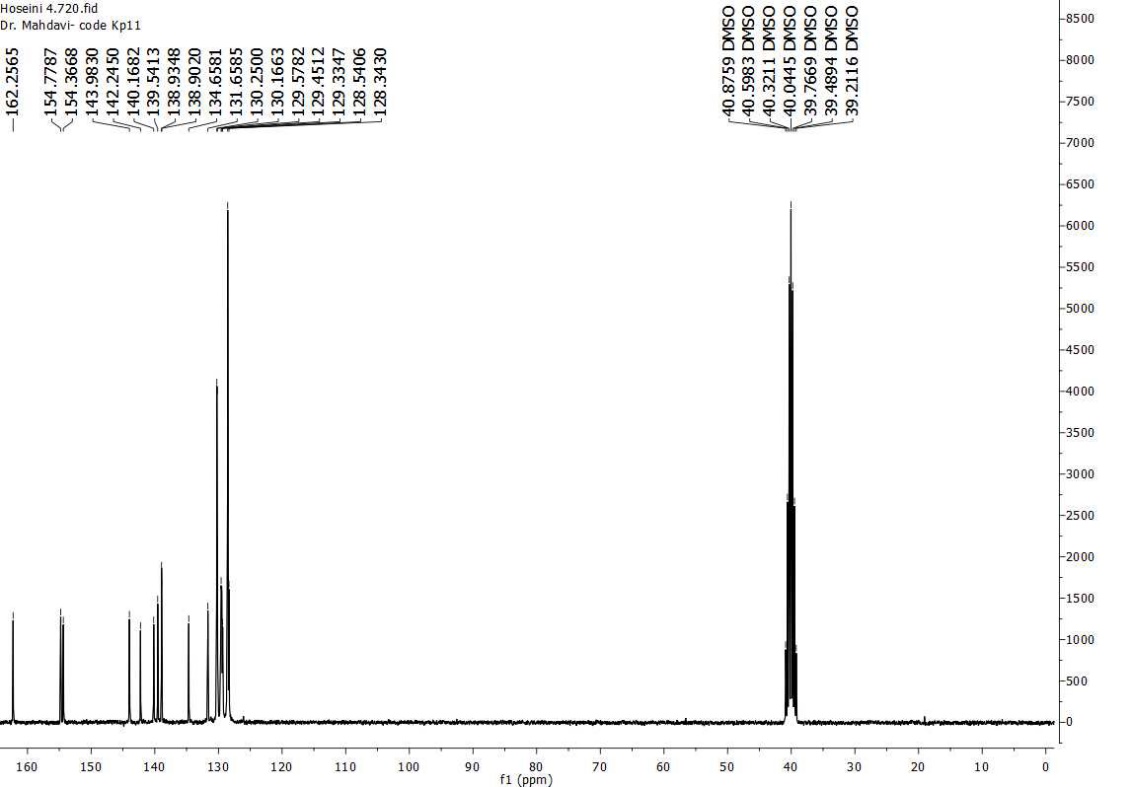
**
